# Modelling quantitative fungicide resistance and breakdown of resistant cultivars: Designing integrated disease management strategies for Septoria of winter wheat

**DOI:** 10.1371/journal.pcbi.1010969

**Published:** 2023-03-28

**Authors:** Nick P. Taylor, Nik J. Cunniffe

**Affiliations:** Department of Plant Sciences, University of Cambridge, Cambridge, United Kingdom; Fundação Getúlio Vargas: Fundacao Getulio Vargas, BRAZIL

## Abstract

Plant pathogens respond to selection pressures exerted by disease management strategies. This can lead to fungicide resistance and/or the breakdown of disease-resistant cultivars, each of which significantly threaten food security. Both fungicide resistance and cultivar breakdown can be characterised as qualitative or quantitative. Qualitative (monogenic) resistance/breakdown involves a step change in the characteristics of the pathogen population with respect to disease control, often caused by a single genetic change. Quantitative (polygenic) resistance/breakdown instead involves multiple genetic changes, each causing a smaller shift in pathogen characteristics, leading to a gradual alteration in the effectiveness of disease control over time. Although resistance/breakdown to many fungicides/cultivars currently in use is quantitative, the overwhelming majority of modelling studies focus on the much simpler case of qualitative resistance. Further, those very few models of quantitative resistance/breakdown which do exist are not fitted to field data. Here we present a model of quantitative resistance/breakdown applied to *Zymoseptoria tritici*, which causes Septoria leaf blotch, the most prevalent disease of wheat worldwide. Our model is fitted to data from field trials in the UK and Denmark. For fungicide resistance, we show that the optimal disease management strategy depends on the timescale of interest. Greater numbers of fungicide applications per year lead to greater selection for resistant strains, although over short timescales this can be oset by the increased control oered by more sprays. However, over longer timescales higher yields are attained using fewer fungicide applications per year. Deployment of disease-resistant cultivars is not only a valuable disease management strategy, but also oers the secondary benefit of protecting fungicide effectiveness by delaying the development of fungicide resistance. However, disease-resistant cultivars themselves erode over time. We show how an integrated disease management strategy with frequent replacement of disease-resistant cultivars can give a large improvement in fungicide durability and yields.

## Author summary

Plant pathogens pose a major threat to crop yields. The two most common forms of disease control, namely use of fungicides and deployment of disease resistant cultivars, are threatened by pathogen evolution causing fungicide resistance or erosion/breakdown of cultivar control. There are two categories of resistance/breakdown; qualitative or quantitative. Although resistance to many cultivars and the most common fungicides is quantitative, the mathematical modelling literature focuses almost exclusively on qualitative resistance, for simplicity or due to lack of appropriate data required to fit a model of quantitative resistance. In this study we present the first model focusing on both quantitative fungicide resistance and cultivar breakdown to be fitted to field data. We use the disease of wheat, Septoria leaf blotch, as a case study. After fitting our model to field trial data from the UK and Denmark, we use it to demonstrate how to design sustainable disease management strategies that optimise yield. We show that combining resistant cultivars with fungicide applications can prolong the effectiveness of both strategies, but that the optimal number of fungicide applications depends on the timescale of interest. Over short timescales, the optimal strategy uses more fungicide applications per year than over longer timescales.

## Introduction

Plant pathogens are a significant threat to global food security [1, 2]. Diseases caused by plant pathogens routinely lead to large losses in crop yields [3], and significant proportion of growers’ time and money can therefore be spent on disease management [4]. Spraying crops with fungicide and using cultivars which are genetically resistant to diseases are two of the most effective control strategies. However, each presents huge selection pressure on pathogen populations, which consequently evolve in response [5]. This leads to pathogen populations which exhibit fungicide resistance [6], as well as the loss of effectiveness (‘breakdown’) of disease resistant host cultivars [7]. Mathematical models have an important role in understanding the processes underlying these changes [8], which involve complex interactions occurring over long time scales.

Both fungicide resistance and breakdown of disease resistant crop cultivars can be characterised as qualitative or quantitative [9, 10]. Qualitative resistance/breakdown is usually caused by a single genetic change leading to a major shift in the characteristics of the pathogen population of interest [11]. Qualitative resistance/breakdown is therefore sometimes called monogenic [12]. For some fungicides, a single genetic change in the pathogen leads to a step change in the fungicide sensitivity of affected individuals. For resistant cultivars, qualitative breakdown tends to occur in cases in which disease resistance depends on a single resistance gene. A single pathogen mutation can then lead to the cultivar suddenly becoming susceptible to disease. For both fungicides and host plant resistance, qualitative resistance/breakdown involves a very sudden transition from good to bad control, even if the control had previously been effective for several years.

In contrast, quantitative resistance/breakdown is characterised by small, gradual shifts in the make-up of the pathogen population, with multiple genetic changes which accumulate over time [9, 10]. For fungicides often these changes are polygenic, although in some cases multiple mutations in the same gene can lead to a gradual loss of effectiveness in control. Reduced susceptibility of modern disease resistant cultivars is often achieved by stacking or pyramiding minor resistance genes (i.e. combining more than one gene for resistance), each with a small effect on disease susceptibility. This means that the pathogen population gradually attains resistance, and since mutations in different avirulence genes are involved, this involves polygenic changes in the pathogen population. In both cases, the typical pattern is control that has partial, but diminishing, effect for several growing seasons until eventually it becomes too ineffective for adequate control in the field.

Quinone-outside inhibitor fungicides (QoIs) provide an example of qualitative (monogenic) fungicide resistance. In particular, the G143A mutation in *cytb* (cytochrome b gene) is linked to high levels of resistance to QoI fungicides that disrupt electron transport during cellular respiration [13]. Taking an example from the important wheat disease Septoria tritici blotch (STB, caused by *Zymoseptoria tritici*), QoIs were very effective when they were first introduced in the 1990s. However, after only a few years of use they became ineffective [14], and the shift from good to poor control happened very rapidly. In 2002, approximately 80% of fungicide sprays contained QoIs in a wheat disease monitoring program in northern Germany [15]. After breakdown of QoI efficacy, their use dropped to around 4% in 2006 [15]. Contemporary fungicide spray programmes targeting control of STB tend to rely upon azole and succinate dehydrogenase inhibitor (SDHI) fungicides [16, 17], to which resistance is quantitative. For azoles, multiple mutations in the CYP51 enzyme are most often implicated [18, 19].

Often growers combine fungicides and resistant cultivars, sometimes as part of so-called ‘integrated disease management’ strategies. The philosophy, closely linked to ‘integrated pest management’ (IPM), emphasises simultaneous deployment of multiple control strategies. The underpinning idea is to obtain more effective control but with less dependence on any single strategy, particularly chemicals [20]. In agriculture in the developed world, growers routinely combine resistant cultivars with fungicide spray programmes. Clearly this has the potential to lead to more effective control in any single season. However, the theory of fungicide resistance indicates any factor causing an overall reduction in pathogen growth rate of both resistant and sensitive pathogen strains leads to slower selection and so an increased lifetime of the chemical of interest [21]. Recent theoretical work has applied this to show how fungicide sprays can also make host resistance more durable [22].

In general, mathematical models have played a central role in understanding how control via fungicides and cultivar resistance can be optimised [8, 23]. Until now, mathematical modelling has primarily focused on qualitative resistance/breakdown [24–32], with very few focusing on quantitative resistance [33–35]. However, resistance to many fungicides and resistant cultivars currently in use is quantitative. Despite the benefits of integrated disease management, few models in the literature address both fungicide and cultivar resistance, with most focusing on either fungicide [25, 26, 28, 36] or cultivar [29, 35, 37, 38] control. In this paper we present a model of quantitative resistance incorporating both fungicide and disease-resistant cultivar control.

In the modelling literature for fungicide resistance, evolution is typically split into an ‘emergence phase’ and a ‘selection phase’. The emergence phase is the period during which resistant strains invade the pathogen population following a mutation event [39]. The selection phase is the period after the emergence phase, in which strains with different fungicide sensitivities are present in the population, and when resistant strains are selected for by fungicide application. The models currently in the literature address either the emergence phase [27] or the selection phase [26, 28]. The model presented in this paper instead represents both phases of resistance development in a single model framework.

Plant disease systems are inherently stochastic, most obviously because of the effects of weather on pathogens’ spread [40]. However, few models of fungicide resistance or cultivar susceptibility incorporate environmental stochasticity, despite some exceptions [41–43]. We aim to understand the impact of environmental stochasticity on the optimal disease management recommendation. Using environmental variability in the model is not only inherently more realistic, but also helps us understand the best- and worst-case scenarios for proposed disease management strategies.

To the best of our knowledge, the model we present here is the first that focuses on quantitative resistance and is fitted to field data for both cultivar and fungicide control. We use STB, the most prevalent disease of wheat worldwide [17, 44], as our case study. It is a particularly economically important disease; the fungicide market in Europe alone is estimated to be more than $2.4bn [17], of which $1.2bn is primarily targeted towards managing STB. Assessments of control efficacy against STB are routinely taken during field trials of new cultivars [45], and in fungicide resistance assessments [14]. Crucially these programs span multiple years, providing us with a source of the type of data we require to fit our model.

In this paper we use a model of quantitative fungicide resistance and breakdown of resistant cultivars to explore the optimal way to combine host and fungicide control strategies to minimise disease impact and maximise yield. The model includes pathogen mutation, initial standing variation in the pathogen population and environmental stochasticity, and is the first model of quantitative resistance in the literature that is fitted to field data. We use our model to address the following questions:

1. How does the number of fungicide applications per year affect resistance development, disease severity and yield over time?
2. How does environmental stochasticity influence the optimal strategy?
3. How does the optimal strategy depend on the time-frame of interest?
4. How can resistant cultivars be optimally combined with fungicide control for an effected integrated disease management approach?

## Materials and methods

### Model structure

Our model tracks the disease caused by, and evolution of, a pathogen population over multiple growing seasons. Within a single growing season, we model the growth of the pathogen population and the loss of healthy tissue. For simplicity, there is no consideration of spatial effects.

The model is an extension of a simple Susceptible-Infected disease model (*S* − *I*) which includes a more detailed description of the pathogen population. This allows us to explore the evolutionary dynamics of the pathogen in greater detail. In particular, we consider the level of resistance to the fungicide as a continuous spectrum. This allows us to explore the effect of using new fungicides in the face of quantitative pathogen evolution and/or disease-resistant cultivars. Initially we focus on fungicide control only, before exploring the effect of both fungicide and cultivar control. A full description of model variables is found in Table 1.

**Table 1:** List of variables used. Many of the variables vary with time *t*. Infection *I* also depends on trait values *k*, *l*. The pathogen infection rate *β* depends on *k*, *l* and *t*. The uncontrolled pathogen infection rate *β*_0_ depends on the disease pressure in a particular year.

Variable (fungicide only model)	Variable (full model)	Description	Equation
*S*(*t*)	*S*(*t*)	Susceptible host tissue	-
*I*(*k*, *t*)	*I*(*k*, *l*, *t*)	Infected host tissue	-
*D*(*t*)	*D*(*t*)	Disease severity	4
*P*(*a*, *b*)	*P*(*a*, *b*)	Probability of mutation from parent *b* to offspring *a*	11
*G*(*k*, *t*)	*G*(*k*, *l*, *t*)	Per capita growth rate	12, 17
*t*	*t*	Time	-
*k*	*k*	Fungicide sensitivity trait	-
−	*l*	Host susceptibility trait	-
*β*_0_	*β*_0_	Pathogen infection rate (no control)	-
*β*(*k*, *t*)	*β*(*k*, *l*, *t*)	Pathogen infection rate (control applied)	8, 16
*C*(*t*)	*C*(*t*)	Fungicide concentration	7
*g*(*t*)	*g*(*t*)	Host growth function	9
Γ(*t*)	Γ(*t*)	Host senescence function	10

#### Single strain model (fungicide only)

We first consider a single pathogen strain, for the purposes of explanation, before introducing the full model. We assign this pathogen strain a ‘fungicide trait value’ *k*, which describes its fitness with respect to the fungicide. The trait value *k* is between 0 and 1, with 0 being fully sensitive (i.e. the pathogen is fully controlled by a fungicide application, at least directly after it is sprayed) and 1 being fully resistant (i.e. completely unaffected by a fungicide application). We use ‘strain *k*’ as a shorthand for ‘the pathogen strain with trait value *k*’.

The density of susceptible host at time *t* is denoted by *S*(*t*), and the density of host tissue infected by strain *k* at time *t* is denoted by *I*(*k*, *t*). The rate of loss of healthy (susceptible) leaf tissue is given by:

$$\begin{array}{c} \frac{dS(t)}{dt}=g\left(t\right)-\Gamma \left(t\right)S\left(t\right)-S\left(t\right)\beta \left(k,t\right)I\left(k,t\right), \end{array}$$ (1)

where *g*(*t*), Γ(*t*) and *β*(*k*, *t*) are host growth, host senescence and the pathogen infection rate respectively, which are all described below.

The rate of increase of host tissue infected by strain *k* is given by:

$$\begin{array}{c} \frac{dI(k,t)}{dt}=S\left(t\right)\beta \left(k,t\right)I\left(k,t\right). \end{array}$$ (2)

We normalise the tissue quantities, which correspond to leaf area, so that the total density of healthy host tissue (*S*) and infected host tissue (*I*) is 1 when host (leaf) growth is complete and before host senescence begins. ‘Disease severity’ (*D*) is the percentage of leaf tissue/leaf area that is infected by any pathogen strain. Define

$$\begin{array}{c} I_{TOT}\left(t\right)=\int_{0}^{1}I\left(k,t\right)dk \end{array}$$ (3)

as the total density of tissue infected by any strain. Although we only have one strain at this stage, we will in general require this integral. Then disease severity is calculated according to:

$$\begin{array}{c} D\left(t\right)=100\frac{I_{TOT}\left(t\right)}{S\left(t\right)+I_{TOT}\left(t\right)}. \end{array}$$ (4)

The pathogen infection rate, *β*(*k*, *t*), depends on the pathogen response to the fungicide. [26, 28, 46] use a fungicide dose response of the form:

$$\begin{array}{c} \epsilon \left(k,t\right)=1-\omega (1-\text{exp}(-\theta (k)C(t))), \end{array}$$ (5)

where *ϵ* is the proportionate effect of the fungicide on strain *k*, *ω* is the asymptote of the dose response curve and *θ*(*k*) is the curvature of the dose response curve with respect to the pathogen, which here also depends on the strain *k*. We choose the asymptote parameter, *ω*, to equal 1, as was used for high risk fungicide in [28]. This means that the infection rate at theoretical infinite dose is 0.

The relationship between *k* and *θ*(*k*) is that *θ*(*k*) = −log(*k*). Then, since we have assumed the asymptote *ω* = 1, we find:

$$\begin{array}{c} \epsilon (k,t)=\text{exp}(\text{log}(k)C(t)). \end{array}$$ (6)

This means that the proportionate effect of the fungicide on the infection rate of this pathogen strain is *k* when the concentration is 1. In general the effect depends on the fungicide concentration, which is assumed to decay exponentially with time *t*, at rate Λ, after each application as in [26, 28]. The fungicide concentration equation is:

$$\frac{\text{d}C\left(t\right)}{\text{d}t}=-\Lambda C,$$ (7)

where *C* is initially 0 but instantaneously increases by 1 every time the fungicide is applied. We choose a decay rate of Λ = 0.5 × (6.91 + 11.1) × 10^−3^ = 9.005 × 10^−3^ degree-days^-1^, which is the average of the two fungicide decay rates in [24, 28]. The decay rates in [24, 28] were based on the half lives reported in [25] and the relation Λ = −log(0.5)/*T*_0.5_, where *T*_0.5_ is the half life. For ‘azoxystrobin’, the half life times reported in [25] were 6.9, 3.6, 4.8 and 1.2 days for UK trial data from Edinburgh 2002, Inverness 2002 and Terrington 2002 and 2003, respectively. We follow [26, 28] in assuming perfect exponential decay in fungicide concentration. In general the decay rate parameter will vary depending on the fungicide.

The fungicide effect acts as a multiplier on the underpinning infection rate *β*_0_. When the concentration is 0, the effect is 1, so when there is no fungicide present all strains behave identically. When the fungicide is applied the infection rates of strains with lower *k* values are suppressed more than that of strains with higher *k* values:

$$\begin{array}{c} \beta \left(k,t\right)=\beta_{0}\epsilon \left(k,t\right). \end{array}$$ (8)

The fungicide can be applied at up to three time points: *T*_1_ (growth stage 32), *T*_2_ (growth stage 39) and *T*_3_ (growth stage 61) [28, 36]. A one treatment programme is applied at *T*_2_, a two treatment programme is applied at *T*_2_ and *T*_3_, a three treatment programme is applied at *T*_1_, *T*_2_ and *T*_3_ (Table 2).

**Table 2:** Times that are required in running the model. Here we use Zadoks’ growth scale [47]. The times *T*_1_, *T*_2_ and *T*_3_ are as in [28, 36]. The start time is growth stage 32, corresponding to the emergence of leaf 3 (*T*_1_). The final time *t*_*end*_ (growth stage 75) is interpolated from the value at growth stage 61 (*T*_*GS*61_ = *T*_3_ = 2066 degree-days) and growth stage 87 (*T*_*GS*87_ = 2900 degree-days) from [28, 36]. This assumes that the temperature remains approximately constant in the late stages of the season. We also round the values to the nearest integer. Growth stage 75 was chosen for the end time because it was the time in Dataset D which links yield to disease severity (Table 3). Any host or pathogen growth before *t*_*start*_ can be scaled into the amount of inoculum *I*_0_ in the model at *t*_*start*_; the procedure used to fit the initial inoculum parameter, *I*_0_, is reported in S1 Text.

Parameter	Growth stage	Time (degree-days)	Purpose
*t*_*start*_ = *T*_1_	32	1456	Application programme: 3; also start time
*T*_2_	39	1700	Application programmes: 1, 2, 3
*T*_3_	61	2066	Application programmes: 2, 3
*t*_*end*_	75	2515	End time
*T*_*GS*61_	61	2066	Start of senescence in [28]
*T*_*GS*87_	87	2900	End time in [28]
*T*_1_	32	1456	Fitting *I*_0_
*t*_*A*_	63	2130	Fitting *I*_0_
*t*_*B*_	71	2387	Fitting *I*_0_
*t*_*end*_	75	2515	Fitting *I*_0_

The host is assumed to grow within the season before a period of senescence. We use the same form for growth and senescence as in [25, 28]. The growth equation is:

$$\begin{array}{c} g(t)=r(1-A(t)), \end{array}$$ (9)

where *A*(*t*) is the total amount of healthy and infected tissue. The value for *r* is 0.0126 degree-days^-1^ as in [24, 28]. We non-dimensionalised so that the maximum leaf area is 1, as in [46]. The initial total quantity of host tissue is *H*_0_ = 0.05/4.1, using the values for initial host area and maximum host area from [24].

The senescence equation, which controls the time-dependent rate at which leaf senescence occurs, is:

$$\begin{array}{cc} \Gamma (t)= & \left\{ \begin{array}{c} 0.005(\frac{t-T_{GS61}}{T_{GS87}-T_{GS61}})+0.1e^{-0.02(T_{GS87}-t)},\;\;\text{if}\;t\geq T_{GS61},\\ 0,\;\;\text{if}\;t<T_{GS61}. \end{array}\right. \end{array}$$ (10)

This is the same function / parameterisation as in [28], and originally derived in [24]. See Table 2.

#### Model accounting for diversity within the pathogen population (fungicide only)

The population model, which accounts for diversity within the pathogen population (Table 1) addresses a continuous spectrum of pathogen strains with trait values (*k*) varying from 0 to 1. It generalises the single strain model to an entire population of different pathogen strains with differing initial densities. Healthy host tissue *S* gets infected by different components of the pathogen population, at different rates when the fungicide is present. We assume that the different strains behave identically in the absence of control (i.e. we neglect modelling fitness costs). Ideally we would incorporate fitness costs into the model, but to do so would require a complete data set describing the infection rates for every different pathogen trait value *k* (i.e. each pathogen strain).

Pathogen mutation is considered, so a small proportion of pathogen offspring has a different trait value to its parent. We assume mutation events occur with probability *p*, and we choose a Gaussian mutation kernel with mutation scale *σ*^2^, following [35]. The model in [35] is of cultivar resistance rather than fungicide resistance, so we assume that mutation for cultivar and fungicide resistant traits acts in the same way. We assume that the rate and scale of mutation are constants, i.e. do not vary through the season and they do not depend on the trait value of the parent. This means that mutation acts in the same way for highly resistant strains as for highly sensitive strains.

For a strain *j*, we describe the probability *P*(*k*, *j*) of its offspring taking trait value *k*:

$$\begin{array}{c} P\left(k,j\right)=\left(1-p\right)\delta \left(k-j\right)+p\frac{1}{\sqrt{2\pi \sigma^{2}}}\text{exp}(\frac{-{\left(k-j\right)}^{2}}{2\sigma^{2}}). \end{array}$$ (11)

Here *δ*(*x*) is the Kronecker delta (which is 1 when *x* = 0 and 0 otherwise). All strains must have trait values within [0, 1], so any offspring that are predicted to take negative values are given trait value 0 and any that are predicted to take values greater than 1 are given trait value 1.

The per capita growth rate of offspring with trait value *k* is given by the integral over all possible parents with trait value *j*:

$$\begin{array}{c} G\left(k,t\right)=\int_{0}^{1}\beta \left(j,t\right)I\left(j,t\right)P\left(k,j\right)dj. \end{array}$$ (12)

The total per capita growth rate of new infection at time *t* is given by the integral over all new strains:

$$\begin{array}{c} \bar{\beta I}\left(t\right)=\int_{0}^{1}G\left(k,t\right)dk=\int_{0}^{1}\int_{0}^{1}\beta \left(j,t\right)I\left(j,t\right)P\left(k,j\right)djdk \end{array}$$ (13)

This means fastest growing strains have relatively higher contribution to new infections.

The *S* equation becomes:

$$\begin{array}{c} \frac{dS(t)}{dt}=g\left(t\right)-\Gamma \left(t\right)S\left(t\right)-S\left(t\right)\bar{\beta I}\left(t\right), \end{array}$$ (14)

The *I* equation, for each strain with trait value *k*, becomes:

$$\begin{array}{c} \frac{dI(k,t)}{dt}=S\left(t\right)G\left(k,t\right)\;\text{for}\;k\in \left[0,1\right]. \end{array}$$ (15)

The total amount of initial inoculum is fixed at the same value each year, although evolutionary changes mean that its strain composition varies over years. This choice means the integral over all pathogen strains equals *I*_0_ × *H*_0_ at the start of each modelled season (*t* = *T*_1_, Table 2), where *H*_0_ is the initial amount of host tissue.

#### Incorporating host plant resistance (full model)

A similar modelling approach can be used to explore how the pathogen population adapts to resistant cultivars. We introduce a ‘host trait’ *l*. The main difference between the host trait and the fungicide trait in the model is that the host protection is assumed to act in the same way throughout the season (no time-variation), whereas fungicide control depends on the fungicide concentration which depends on time. The value of *k* is as before, whereas the value of *l* corresponds to the host effect at any time:

$$\begin{array}{c} \beta \left(k,l,t\right)=\beta_{0}l\text{exp}\left(\text{log}\left(k\right)C\left(t\right)\right). \end{array}$$ (16)

Higher host trait values give higher infection rates corresponding to increased pathogen fitness (similarly to the fungicide trait values).

Mutation is assumed to act in a similar way to before, with both *k* and *l* changing, again on a [0, 1] scale. The per capita growth rate of the pathogen strain with fungicide trait *k* and host trait *l* is given by the integral over all possible parent strains with fungicide trait *j* and host trait *m*:

$$\begin{array}{c} G\left(k,l,t\right)=\int_{0}^{1}\int_{0}^{1}\beta \left(j,m,t\right)I\left(j,m,t\right)P\left(k,j\right)P\left(l,m\right)djdm. \end{array}$$ (17)

Host and fungicide mutations are assumed to act independently of each other. This assumption should hold for host/fungicide combinations where different genes control fitness relative to the host/fungicide.

The total per capita growth rate of infection is given by:

$$\begin{array}{c} \bar{\beta I}\left(t\right)=\int_{0}^{1}\int_{0}^{1}G\left(k,l,t\right)dkdl. \end{array}$$ (18)

The model equations become:

$$\frac{dS(t)}{dt}=g\left(t\right)-\Gamma \left(t\right)S\left(t\right)-S\left(t\right)\bar{\beta I}\left(t\right),$$ (19)

$$\frac{dI(k,l,t)}{dt}=S(t)G(k,l,t).$$ (20)

#### Notes on model implementation

When solving the model, we discretise the integral $\bar{\beta I}$ in Eqs 14 and 19. We use at least 100 values for *k* (and *l* if using the full model) in each model run. The ‘discretisation number’ is specified in the parameter values in each figure caption, denoted by *n*_*k*_ for the number of *k* values (and *n*_*l*_ for the number of *l* values if applicable). We typically chose larger values for *n*_*k*_ when using the fungicide only model, since the full model was slower for large values of *n*_*k*_ and *n*_*l*_ (because the discretisation was then over a grid of *n*_*k*_ × *n*_*l*_ values rather than a one dimensional spectrum of *n*_*k*_ values).

The fungicide concentration equation (Eq 7) can be solved for each spray programme and written explicitly. For example, the concentration of a 3 spray programme is given by:

$$\begin{array}{c} C\left(t\right)=f_{1}\left(t\right)e^{-(t-T_{1})}+f_{2}\left(t\right)e^{-(t-T_{2})}+f_{3}\left(t\right)e^{-(t-T_{3})}, \end{array}$$ (21)

where

$$\begin{array}{cc} f_{i}\left(t\right)= & \left\{ \begin{array}{c} 0,\;\;\text{if}\;t<T_{i},\\ 1,\;\;\text{if}\;t\geq T_{i}. \end{array}\right. \end{array}$$ (22)

The form is similar for the 1 and 2 spray programmes, except the $f_{1}(t){\text{e}}^{-(t-T_{1})}$ term is omitted for the 1 and 2 spray programmes and the $f_{3}(t){\text{e}}^{-(t-T_{3})}$ is omitted for the 1 spray programme. In our implementation of the model we use this form rather than solving the differential equation each time the model is run.

### Model fitting

#### Initial inoculum

We fitted the initial amount of inoculum at the start of each season using Dataset A (see Table 3 and also S1 Text). We assumed this initial level was constant over seasons, with differences in disease pressure from season to season captured entirely by the infection rate (*β*_0_), which varies over seasons in our model. The fitted value of *I*_0_ was used for all model runs unless stated otherwise. Dataset A was collected in Denmark from seven locations across three years. The trials reported in Dataset A were conducted by Aarhus University and the knowledge centre for agriculture, SEGES.

**Table 3:** Datasets used in model fitting.

Dataset	Description	Model parameters fitted
A	STB severity at different times within the season (Denmark)	Inoculum *I*_0_
B	Cultivar trial data (Denmark, 1996–2017)	Infection rate *β*_0_, host distribution mean and shape parameters
C	Treated and untreated (by fungicide) STB severity data (UK, 2001–2018)	Fungicide distribution mean and shape parameters, mutation scale upper bound
D	Yield vs STB severity at GS75 (Soenderborg, 2019)	Yield-severity relationship

#### Infection rate

To find the distribution of infection rates *β*_0_, we used data from cultivar trials which were run from 1996 until 2017 (Dataset B). The trials from Dataset B were conducted by the knowledge centre for agriculture, SEGES and the Danish Institute of Technology. The data set includes data about disease severity at growth stage 75 on untreated plots, depending on location and cultivar.

We filtered the raw trial data to consider plots that were untreated by fungicide and excluding those with cultivar mixtures. This is because we were interested in the level of disease in the absence of control measures. We also filtered to only include high pressure locations (higher than 6 on a 1–10 scale), because we were most interested in testing disease management strategies in the regions where disease is the most damaging. Effective disease management is even more important in these locations than in lower disease pressure locations. Note that due to environmental variation, the high disease pressure locations contained a wide range of disease severities from very low values in some years/locations to very high values in others.

We grouped the data by location, cultivar and year, before finding the mean severity score for each location/cultivar/year combination. For each year/location, we found the worst performing cultivar and assumed that this mean severity is representative of the average severity in the absence of control (i.e. that the pathogen has reached peak fitness for that cultivar). We chose ‘Mariboss’ as the disease-resistant cultivar to analyse. It was one of only 4 cultivars which appeared for 10 or more years in the dataset, and it offered a relatively good level of control initially. We filtered the dataset to only include location/year combinations where ‘Mariboss’ was grown. All data for the worst performing cultivar (or cultivars if multiple cultivars had an equally poor average) in each year and location that ‘Mariboss’ was found were used as the values for severity in the absence of control.

We found that a truncated exponential was a good fit to these severity data. The probability density function of the truncated exponential is:

$$\begin{array}{cc} f(x)= & \left\{ \begin{array}{c} \frac{\lambda e^{-\lambda x}}{1-e^{-100\lambda }},\;\;\text{if}\;x\in \left[0,100\right],\\ 0,\;\;\text{otherwise.} \end{array}\right. \end{array}$$ (23)

Fitting the truncated exponential to the data allowed us to sample many values for final disease severities in the absence of control, which we used to generate infection rate (*β*_0_) values. The fitted value of λ is in Table 4. This was done by numerically solving the ODEs using the value for *I*_0_ as found above. For each severity, we used a numerical optimiser (‘minimize’ from the open-source Python package ‘scipy.optimise’) to find the corresponding value of *β*_0_. Since this fit was for the absence of control, it meant the entire population behaved like a single pathogen strain with infection rate *β*_0_. This meant that the output had no *k* or *l* dependence, so the initial host and fungicide distributions and the mutation parameters had no effect on the output. Therefore we only needed the host growth/senescence parameters already described and the value for *I*_0_ already found.

**Table 4:** Parameter values used in the model (to 3 significant figures). The parameter values were found in the model fitting process using the data described in Table 3. The sampled infection rates are generated from a truncated exponential distribution with maximum 100 and parameter λ (see Eq 23).

Parameter	Symbol	Range/Value	Dataset/citation	Figure
Initial inoculum	*I*_0_	0.00986	A	S1 Text, Fig 1
STB truncated exponential parameter	λ	0.0329	B	Fig 2A
Infection rate	*β*_0_	Sampled	B	Fig 2B
Median infection rate	*β*_0,*M*_	0.00787	B	Fig 2B
Mutation proportion	*p*_*M*_	1.23 × 10^−5^	[48]	-
Mutation scale, upper bound	*σ* ^2^	0.0198	C	S2 Text, Fig 1
Fungicide distribution mean	*μ*_*F*_	9.44	C	Fig 2D
Fungicide distribution scale parameter	*s*_*F*_	1.19	C	Fig 2D
Host distribution mean	*μ*_*H*_	0.809	B	Fig 5A
Host distribution mean shape parameter	*b*_*H*_	6.59	B	Fig 5A

#### Mutation

The mutation kernel has two parameters, one for the proportion of offspring which have a different trait value and one for the scale of mutation, which affects how different these trait values are (Eq 11). We assume that the proportion and scales of mutation are the same for fungicide and host traits. We had no data to justify an alternative choice, but reassuringly find via a sensitivity scan that changing the mutation proportion and then re-fitting the model has no effect on the model results, and neither does excluding mutation altogether (S3 Text).

The value for the mutation proportion we use is based on estimates for the total number of spores generated in a growing season and the number of spores per season carrying adaptive mutations for fungicides and resistant cultivars [48]. See S2 Text.

To identify an appropriate value for the mutation scale, we first find an upper bound for it. In general, the loss in control efficacy is caused by a combination of selection on strains that are already present in the population as well as on strains that arise due to mutation. This means we can find an upper bound from the value for mutation scale for which the loss in efficacy could be attributed to mutation alone with no initial standing variation (S2 Text). We then arbitrarily choose 10% of this value to the be the value for the mutation scale used in the model, but we test this assumption in S3 Text and find that the model output is very similar for a wide range of choices of mutation scale.

#### Fungicide trait distribution

We fitted an initial distribution for the fungicide trait to reflect the fact there is some initial variation in pathogen fitness relative to the fungicide. To find this distribution we used information about control efficacy derived from disease severity data (Dataset C) from UK disease trials from 2001–2018 [14, 49]. The level of initial control in the data is linked to the mean of the pathogen distribution, because the average pathogen trait value determines how effective control is. The rate of decline in control in the data is linked to the variance of the pathogen distribution, because more variance in the pathogen population leads to a faster breakdown in control. [49] fitted curves to these data, of the form

$$\begin{array}{c} S_{rel}=1-R_{D}+R_{D}\text{exp}\left(-\kappa D\right), \end{array}$$ (24)

to 5 different dosages *D*, where *S*_*rel*_ is related to control by control = 100 − 100*S*_*rel*_, so that control takes values between 0% and 100%. They reported the mean value and standard error for the two parameters *R*_*D*_ and *κ* in each year, but did not report the actual control values (nor the distribution of control values). The parameter *R*_*D*_ relates to the maximum reduction in severity at theoretical infinite dose and *κ* is a curvature parameter.

We used a t-distribution [50] with 3 degrees of freedom to drive a procedure based on the reported standard errors to sample 500 values per year for *R*_*D*_ and *κ*. We then used these values to generate 500 control values each year for use when fitting the model (which helps give an idea of the uncertainty in the measurements and therefore a better measure of model performance). We used a t-distribution because the sample sizes were small and the true standard deviation of the parameters was unknown. We filtered out any generated control values less than 0 and greater than 100 and resampled to replace them until we had 500 values in every year. The bias introduced by this filtering process was small, changing the mean value by 0.83% on average and 2.3% at most across the 18 years in the dataset. Using the uncertainty rather than just a single fitted value per year allowed a clearer assessment of how the model performed on the training and the test set given the variability in the data, and meant that years with lower uncertainty would have a larger influence on the optimal model parameters found in the model fitting process.

We use a gamma distribution to characterise the initial distribution of fungicide curvature parameter, *θ* = −log(*k*). The curvature parameter *θ* takes values in (0, ∞). The gamma distribution is a continuous, two parameter distribution with support on (0, ∞). We use the shape and rate parameterisation. It provides a flexible distribution with support on the appropriate range taken by *θ*. This allows us to compare the mean of the fitted distribution for *θ* with those found in the literature in [26, 28, 46]. We found the optimal values for the two-parameter gamma distribution (i.e. the values that minimised the sum of squared distance between the data and the model output) using the open-source Python package Optuna (v2.10.0) to find the optimal values, sampling our search-space using their implementation of a so-called ‘tree-structured Parzen estimator’ (see [51]), which is more efficient than random sampling since it uses information from previous samples (i.e. a Bayesian approach) to reduce the time spent evaluating ‘bad’ hyperparameters.

The default untreated disease severity reported by [49] was 37%, corresponding to the top 10% of disease severities measured in untreated plots, averaged across all years of trials. [49] did not report a separate value for each year nor standard error terms, so we used this single value for the disease severity in the absence of control in each year. The cultivars used in the experiment were selected for their susceptibility to septoria [49], so we assume that they offer negligable levels of control. We used an infection rate (*β*) value corresponding to the 37% value for every year.

We split our data into a training and a test set, using 2/3 of the data by year for the training set (the training set was from 2001–2012, and the test set from 2013–2018).

#### Host trait distribution

In each year and location (Dataset C), we found the level of control offered by the disease-resistant cultivar ‘Mariboss’ relative to the worst performing cultivar, using the following formula:

$$\begin{array}{c} C_{y,L}=100\times (1-\frac{S_{M}}{S_{W}}), \end{array}$$ (25)

where *S*_*M*_ is the mean severity for ‘Mariboss’ and *S*_*W*_ is the mean severity for the worst cultivar in that particular year *y* and location *L*, where severity is measured on leaf 2.

We use a beta distribution to describe the initial distribution of host trait values *l*. Beta distributions are continuous, two parameter distributions defined on the range (0, 1). They take a wide variety of shapes and commonly appear in population genetics contexts [52], and have support on the appropriate parameter range for *l*: (0, 1). The host trait value *l* is defined on this range and so the beta distribution was an appropriate choice (in contrast to the fungicide trait parameter, for which a positive value was the only constraint since we fit a distribution of curvature values *θ* rather than trait values *k*).

Again we use Optuna to optimise the values of the two distribution parameters, allowing us to find the pair of values for these parameters which gave the best fit between the model output and the data. This was characterised in terms of a loss function L (Eq 27), which was the squared distance between the model and the data, weighted so that the control values that were informed by more data points carried greater importance to the model fit.

Let *M*_*y*_ be the control prediction from the model with no fungicide applications in year *y*, i.e. the control value obtained from comparing the disease severity (*S*_*N*_) with no control (so *β*(*k*, *l*, *t*) = *β*_0_ for all *k*, *l*, *t*), and the disease severity (*S*_*C*_) with cultivar control but not fungicide control (so *β*(*k*, *l*, *t*) = *β*_0_*l* for all *k*, *l*, *t*). Then *M*_*y*_ is given by:

$$\begin{array}{c} M_{y}=100(1-\frac{S_{N}}{S_{C}}), \end{array}$$ (26)

analogously to how *C*_*y*,*L*_ was calculated (Eq 25).

The cultivar control observed in the data in year *y* and location *L* is *C*_*y*,*L*_. We have multiple locations in each year, up to *N*_*y*_ in year *y*. Then let *n*_*y*,*L*_ denote the minimum of the number of trials from the worst performing cultivar and of ‘Mariboss’ in the each year/location combination.

$$\begin{array}{c} L=\sum_{y=2007}^{2016}\sum_{L=1}^{N_{y}}n_{y,L}{\left(M_{y}-C_{y,L}\right)}^{2}. \end{array}$$ (27)

Again we separate the data into a training and test set with a 2/3 split by year (the training set was 2007–2012, and the test set was 2013–2016).

#### Yield

To analyse the relationship between disease severity and yield, we used trial data from Soenderborg (Dataset D), a high disease pressure region of southern Denmark. The data were collected in 2019, and reports disease severity on leaf 2 and yield for a variety of cultivars, including ‘Sheriff’, ‘Kalmar’ and ‘Informer’, meaning that our yield relationship is effectively averaged across different cultivars. Although we expect that yield will vary depending on the cultivar, the dataset was too small to further restrict it to a single choice of cultivar. The dataset corresponds to trials from Soenderborg, but we assume that the response of wheat yields to a particular level of disease is the same regardless of location. Rather than arbitrarily choose a functional form to model the yield-severity relationship, we used a generalised additive model (GAM) to find a smooth curve relating yield and severity as determined by the data. We used the open-source Python package ‘pyGAM’ to fit a monotonically decreasing GAM with 5 splines. The choice of 5 splines was arbitrary, but chosen so that there were enough splines to fit the data well but not so many that the model was overfitting. See S1 Data Data for a CSV file relating disease severity values to yield values.

## Results

### Basic behaviour of the model of fungicide resistance

**Fig 1:**
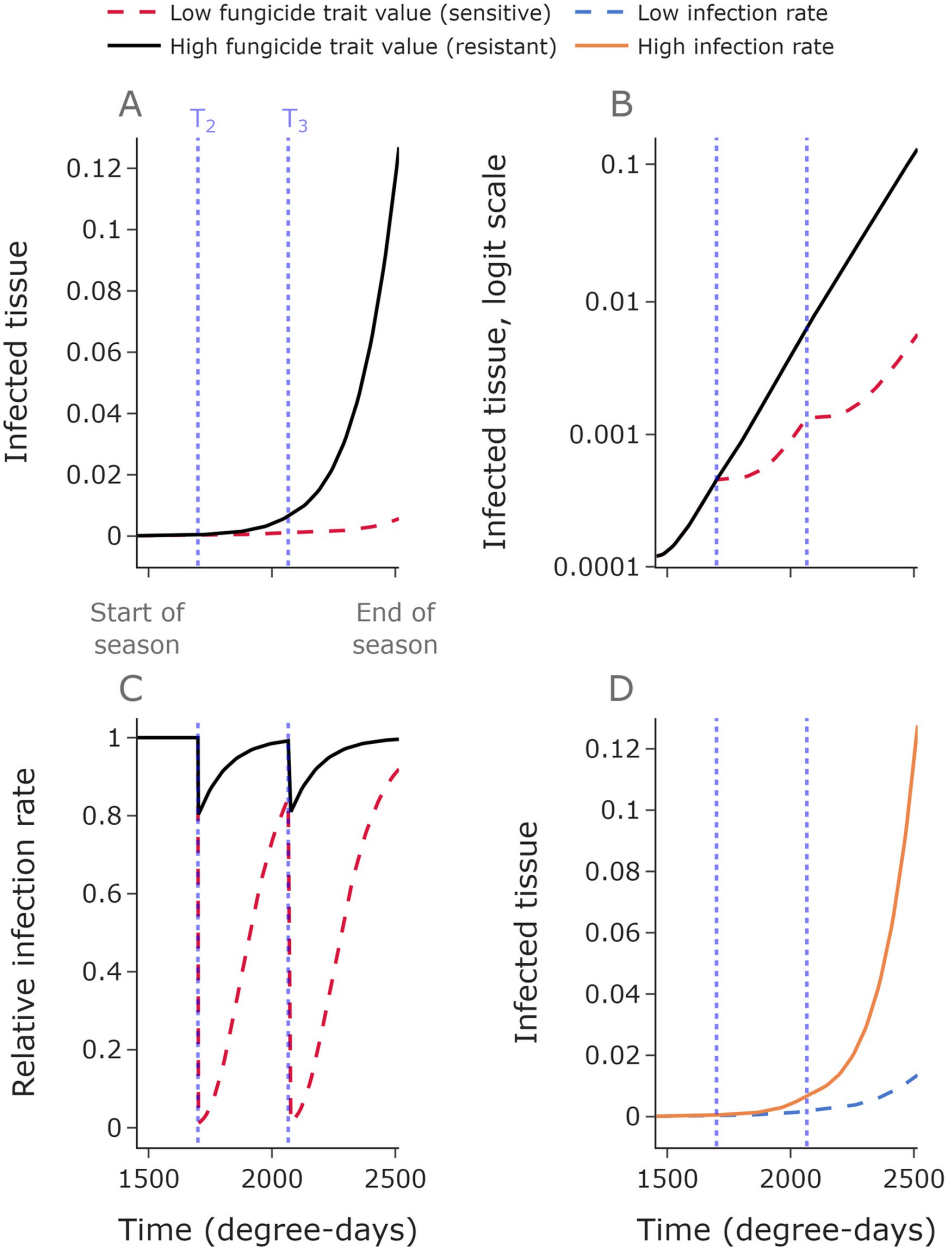
The density of infected tissue depends on the degree of fungicide resistance and infection rate. The dotted blue lines show the two fungicide application times used here. The density of infected tissue is much greater for a pathogen strain with a high fungicide trait value (i.e. more resistant) than for one with a low fungicide trait value (i.e. more sensitive)—see panels **A,B**. Panel **B** shows the same disease progress curves on a logit scale (if *p* ∈ (0; 1) is the density of infected tissue, then its logit is logit(*p*) = log_10_(*p*/1 − *p*)), and shows how disease progression is approximately logistic (i.e. linear on a logit scale). Panel **C** shows how the relative infection rate varies depending on the fungicide trait values and as the fungicide concentration decays following applications. The baseline infection rate *β*_0_ affects the disease progress curve; high *β*_0_ values give greater infected tissue than low *β*_0_ values (**D**). **Parameter values: A,B,C**: *β*_0_ = 0.008 degree-days^-1^, *I*_0_ = 0:01, low trait value = 0.01, low trait value = 0.8. **D**: low *β*_0_ = 0.006 degree-days^-1^, high *β*_0_ = 0.009 degree-days^-1^, *I*_0_ = 0.01, trait value = 0.4. The mutation proportion in all panels is 0. Here *n*_*k*_ = 100.

Initially we consider the case where control is offered by the fungicide only, and a susceptible cultivar is used. During the season, the disease severity (total level of disease) increases. Different strains within the pathogen population grow at different rates depending on their fitness relative to the fungicide (Fig 1A). The increase of disease is approximately logistic when the fungicide is not present (Fig 1B). Strains with greater fitness relative to the fungicide are slowed to a lesser extent by each fungicide application.

When present, the fungicide acts to reduce the infection rate by an amount which depends on the pathogen strain (Fig 1C) and on the concentration of chemical, which decays exponentially after each fungicide application. Disease pressure varies by year; this can be represented in the model by changing the value of the infection rate *β*_0_ in each simulated year (Fig 1D).

### Model fitting

**Fig 2:**
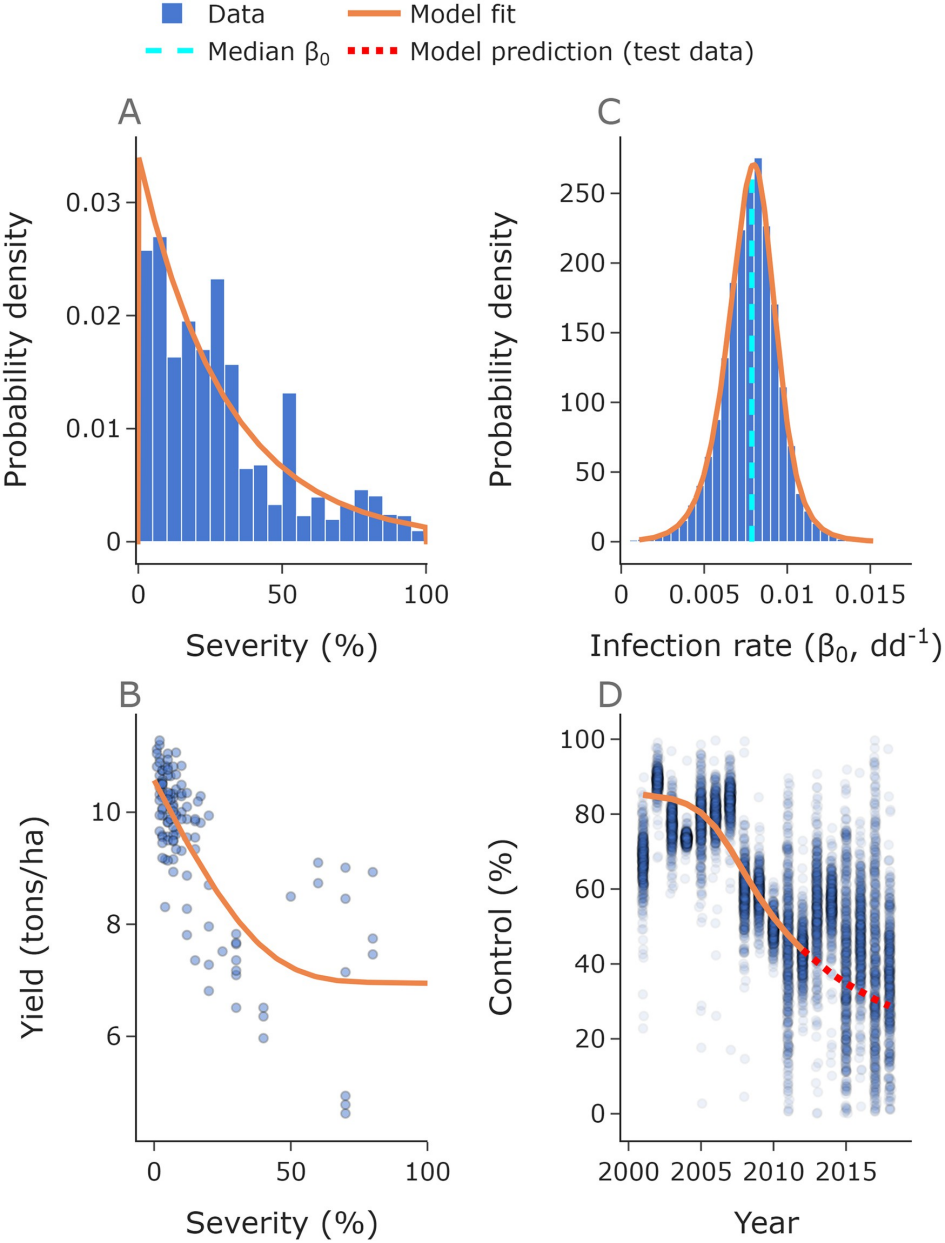
Model fitting. We fitted a truncated exponential to the severity data, allowing us to sample as many random values for disease severity as we need for each simulation (**A**). From these severities and the fitted value for inoculum *I*_0_ we can find a probability density function for the infection rate *β*_0_, shown here alongside a histogram of values inferred from the observed severity data (**B**). Here ‘dd^-1^’ is degree-days^-1^. We used a generalised additive model to describe the relationship between yield and severity (**C**). We used fungicide control data from 2001 to 2012 (solid line) to find the optimal initial trait distribution parameters and used data from 2013 to 2018 (dotted line) as a test set **D**. The mean squared residual on the training set was 108.4 and on the test set was 377.6 (see S3 Text, Table 1). Here *n*_*k*_ = 400.

The truncated exponential fits the disease severity data and was used to sample many values for final disease severities (Fig 2A, Table 4). The infection rate (*β*_0_) values generated from the observed data are plotted in Fig 2B, along with the probability density function for *β*_0_ generated from the truncated exponential distribution for disease severities. The result of the fitted GAM used for the yield-severity relationship was a smoothly varying curve through the data (Fig 2C). The optimal model fit, including mutation parameters and initial trait distribution leads to a control curve passing through the data, including extrapolation to ‘test’ data not used as ‘training’ data for fitting the model (i.e. years past 2012, Fig 2D).

### Model outputs

#### Comparing numbers of fungicide applications

**Fig 3:**
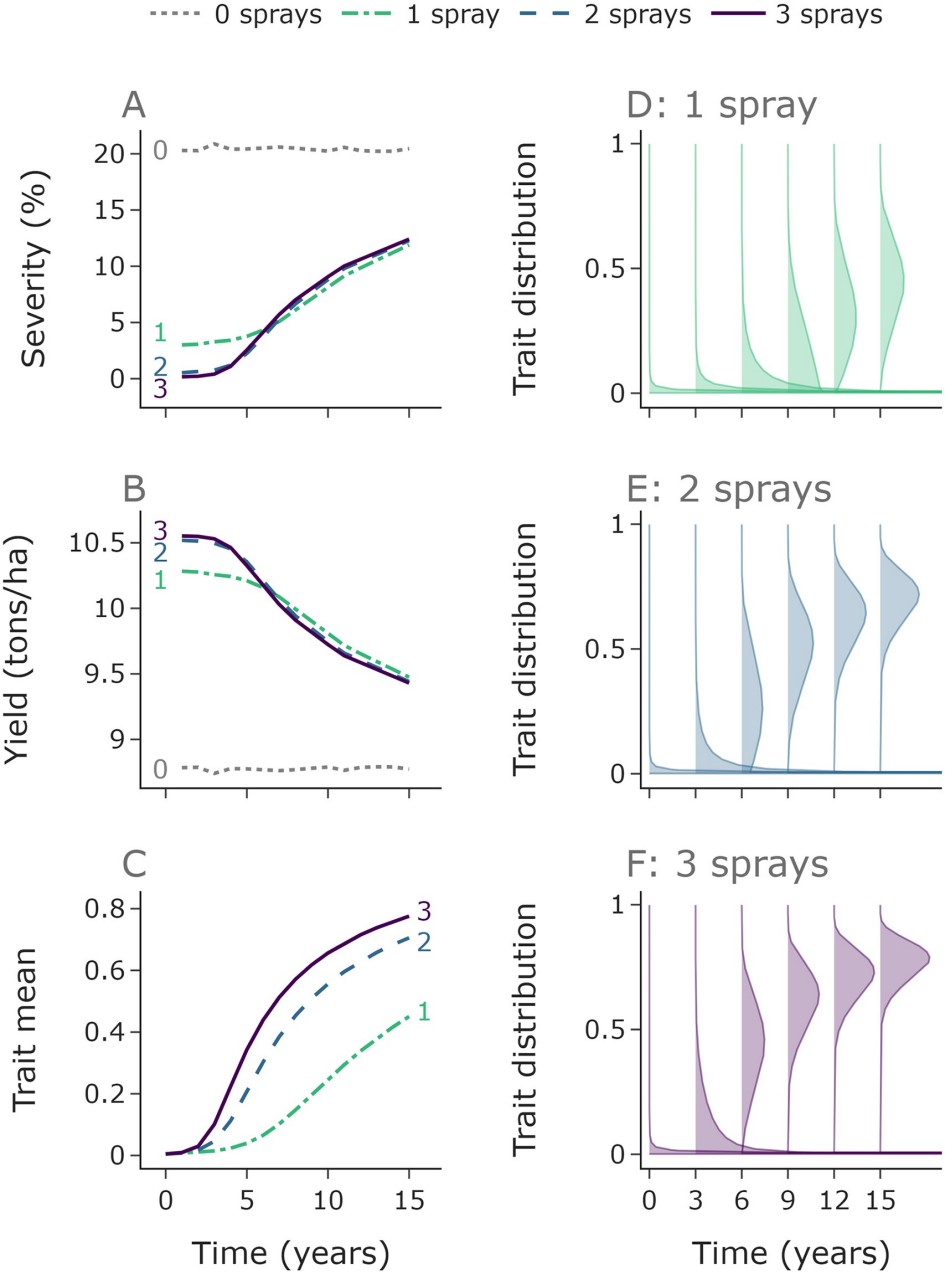
More fungicide applications per year leads to faster selection for resistant strains and to lower yield in the long term. Median values for disease severity and yield from each year of the 10,000 simulations (**A**, **B**). Initially three fungicide applications per year gives the lowest severity and highest yield. Panels **C**-**F** relate to the ‘mean distribution’ across the 10,000 ensembles, and the mean value of this mean distribution in **C**. Due to more rapid selection for resistant strains with more fungicide applications (**C**-**F**), the one fungicide application per year strategy outperforms the two and three applications per year strategies in terms of both severity and yield by year 7. Here *n*_*k*_ = 500.

**Fig 4:**
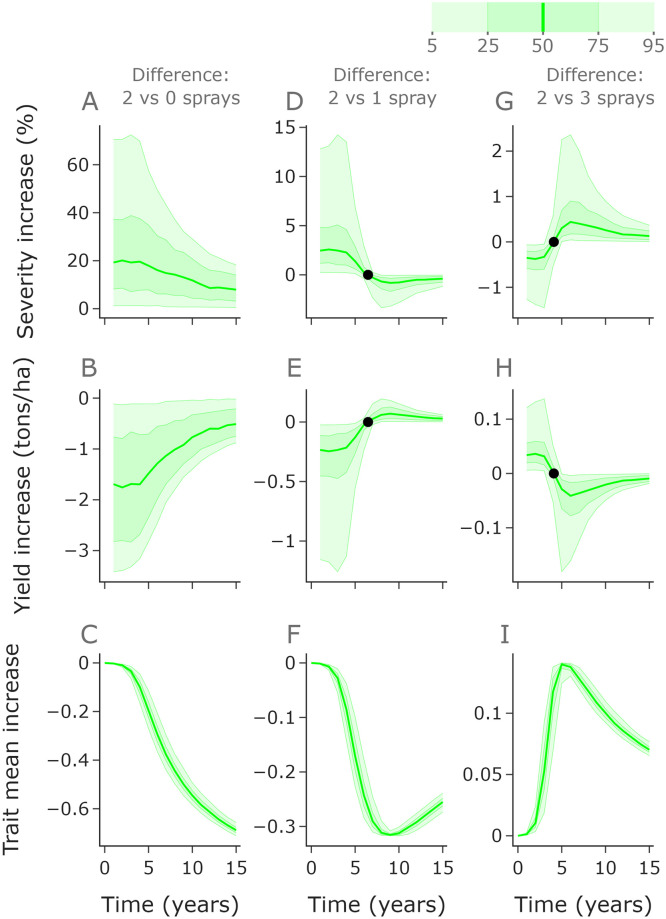
The optimal disease management strategy depends on the relevant timeframe. In the left, middle and right columns we compare the performance of zero, one and three fungicide applications annually with the performance of two applications annually. Zero applications (no control) leads to higher severity (**A**) and lower yield (**B**) than two applications, although the difference reduces over time due to selection for resistant strains (**C**). Initially one application leads to increased severity (**D**) and decreased yield (**E**), but by year 7 one application is better than two due to reduced selection for resistant strains (**F**). Conversely, three fungicide applications initially leads to lower severity (**G**) and higher yield (**H**), but by year 5 this benefit is lost due to increased resistance (**I**). The black dots (panels **D**,**E**,**G**,**H**) denote when the median line crosses 0, i.e. when severity/yield changes so that 1 spray outperforms 2 / 2 sprays outperform 3. Here *n*_*k*_ = 400.

We explore the effect of the number of fungicide applications per year on the pathogen population, disease severity and yield. The results shown in Figs 3 and 4 do not include disease-resistant cultivar control and consider the effect of fungicide applications only. Fig 3 shows the average result across an ensemble of runs, whereas Fig 4 shows the variability in outcomes from the ensemble.

Initially, a greater number of applications offers greater control than fewer (Fig 3A). The figure shows the median value for disease severity and yield in each year over an ensemble of 10,000 runs, each one using randomly sampled values (from our fitted distribution) for the infection rate *β*_0_. It shows the mean density of the distribution at each discretised value for *k*, resulting in an ‘average’ distribution across the ensemble (Fig 3C, 3D, 3E and 3F). The strategies with more fungicide applications lead to faster selection for higher trait values, leading to a gradual shift in distribution (Fig 3D, 3E and 3F). This faster shift in distribution leads to the order in terms of disease severity and yield reversing such that 1 fungicide application per year outperforms 2 and 3 applications per year (Fig 3A and 3B). The higher the number of applications, the more rapidly the distributions shift, which can be shown most clearly by looking at the mean of the distributions (Fig 3C).

Initially the control offered by just one application is strong. This means that the improvement in severity and yield offered by further applications is marginal, with two and three applications performing comparably initially. Their performance remains similar in subsequent years due to a trade off—more applications means greater amount of time with fungicide control, but also faster selection for resistance. This trade off leads to two and three strategies behaving similarly, whereas by year 7 the reduced selection from the one fungicide application strategy outweighs the reduced control caused by fewer applications, and after year 7 the single fungicide application programme performs optimally for yield.

In the early years almost all of the pathogen density is found close to *k* = 0, i.e. highly sensitive strains. Due to strong selection (and also mutation) the distributions broaden over time (Fig 3D, 3E and 3F). Eventually, for 2 or 3 sprays, the distributions narrow around the highly resistant strains with high *k* values (Fig 3E and 3F). Given more years, this narrowing also occurs for a 1 spray programme. These distribution changes are caused by strong selection for highly resistant strains which are at very low density (or arise due to mutation). As these strains outcompete more sensitive strains, we find a range of pathogen strains coexisting briefly, but eventually the sensitive strains are so strongly suppressed that (given enough time/enough sprays) the population comprises almost exclusively of highly resistant strains (e.g. Fig 3F). Note that the distributions approximately match for the same number of sprays. For example, the 1 spray programme distribution looks very similar after 6 years as a 2 spray programme after 3 years, at which point both populations have been subjected to 6 fungicide applications (Fig 3D and 3E).

#### Ranges of outcomes under different numbers of fungicide applications

To further explore the relative performance of differing numbers of fungicide applications, we compare the full distribution of outcomes under zero, one and three applications per year to two applications (Fig 4). We use 1,000 simulations with infection rates sampled randomly from our fitted distribution from Fig 2B. The same set of sampled infection rates were used for each application strategy.

Zero applications per year (no treatment) gives higher severity and lower yield (Fig 4A and 4B) than two applications per year, with particularly extreme differences in the early years whilst the fungicide still offers good control in the two application programme. Within 15 years the fungicide loses efficacy due to the shift in pathogen distribution (Fig 4C), meaning that the control offered is reduced.

One application per year initially gives higher severity and lower yield (Fig 4D and 4E) than two application per year, but by year 7 the median yield is higher and severity is lower due to two applications leading to higher levels of resistance (Fig 4F), as indicated by the summarised results in Fig 3.

Three applications per year initially reduces severity and increases yield relative to two (Fig 4G and 4H). However, by year 5 the increased resistance in the pathogen population (Fig 4I) leads to two applications outperforming three. Interestingly the yield difference in high disease pressure years (95th percentile) in years 5 and 6 are more extreme than the differences in the early years. That means that the more rapid increase in resistance from using 3 sprays can cause bigger yield losses than the improvement in control when the population is highly sensitive. The differences in performance are less exaggerated for the three versus two comparison than for the one versus two or zero versus two comparisons. This is because the difference in control (at least initially) between two and three sprays is smaller than between one and two, which is smaller than between zero and two.

Note that the variation in trait mean is smaller than in yield and severity (Fig 4). Selection increases as disease pressure (and correspondingly severity) increases. However, this increase is much smaller than the difference obtained by extra sprays (e.g. in Fig 4F and 4I the difference from 0 is much bigger than the width of the uncertainty bands). Interestingly in the one versus two and two versus three spray comparison, the difference in trait mean reduces eventually (Fig 4F and 4I). This is because the pathogen distributions eventually approach a trait mean of 1 for all of the strategies, excluding the zero spray strategy (see Fig 3D, 3E and 3F).

#### Introducing disease-resistant cultivars

**Fig 5:**
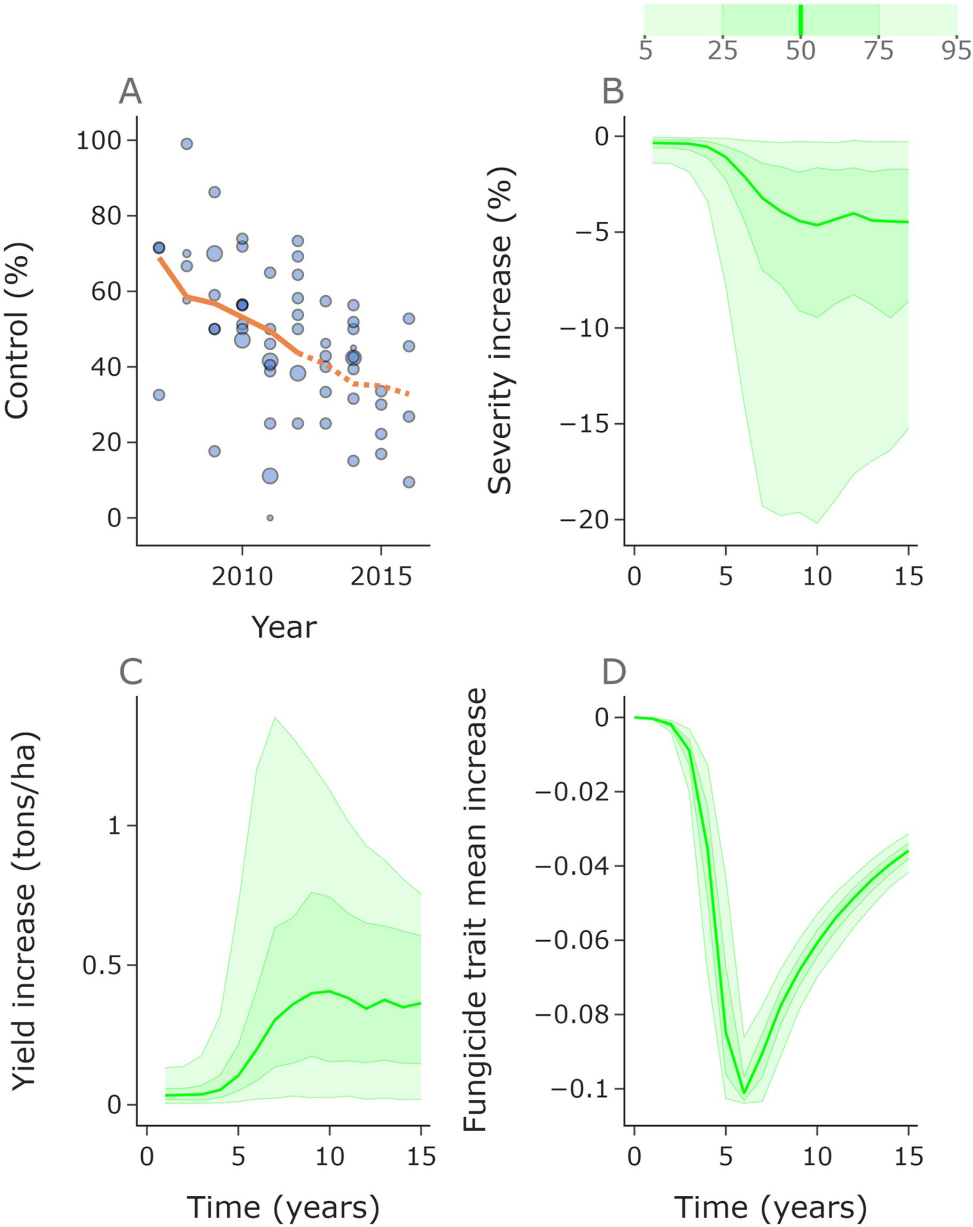
Disease-resistant cultivars reduce severity and protect the fungicide. The optimal host trait initial distribution parameters were fitted using data from 2007 to 2012 (solid line, **A**). We used data from 2013 to 2016 as a test set (dotted line). When comparing a two application fungicide programme with and without the disease-resistant cultivar, the disease-resistant cultivar reduces the severity (**B**) and increases yield (**C**); in each case the response shown is the difference between two spray fungicide programmes with and without the resistant cultivar. Further, the fungicide develops resistance more slowly due to reduced disease pressure when the resistant cultivar is used (**D**). This means the benefit of using the resistant cultivar becomes greater as time goes on, and is particularly noticeable in high disease pressure years with yield improvements of up to 1.39 tons/ha in the 95th percentile in year 7. Here *n*_*l*_, *n*_*k*_ = 100.

Fig 5 shows the results of introducing disease-resistant cultivar control, comparing the results of a two-spray programme with and without host-plant protection from the disease-resistant cultivar ‘Mariboss’. The model fit for ‘Mariboss’ is shown in Fig 5A, corresponding to the model run using the initial trait parameters. The optimal trait parameters are the ones which best matched the data showing decline in cultivar control in the absence of fungicide treatment. The disease-resistant cultivar helps reduce disease severity and increase yield (Fig 5B and 5C). The use of the resistant cultivar reduces the disease pressure, which protects the fungicide, as shown by the difference in fungicide trait mean between the strategies (Fig 5D). This protection offered by the host to the fungicide means that although the difference in yield and severity in year 1 is small, by year 7 the improvement in yield is much larger, particularly in high disease pressure years. This is because the control offered by the fungicide alone is excellent initially, so both strategies offer excellent control and the difference in yield is minimal. However, in later years the benefit of choosing a resistant cultivar becomes significant—greater than the initial benefit of using 2 sprays rather than 1 or 3 rather than 2 in Fig 4E and 4H.

#### Varying host control and regular replacement of disease-resistant cultivars

To further explore the extent to which a disease-resistant cultivar protects the fungicide (and vice versa), we ran a parameter scan testing the performance of different hypothetical cultivars. We were interested to determine how changes in cultivar efficacy would impact the rate of fungicide breakdown. We allowed the initial mean of the host trait distribution to vary whilst keeping the shape parameter of the distribution fixed at the value from the model fitting. Some examples of the resulting distributions are shown in S4 Text. For simplicity, we used the median value *β*_0_ = *β*_0,*M*_ (Table 4) in all examples, i.e. the underlying simulations did not account for environmental stochasticity.

**Fig 6:**
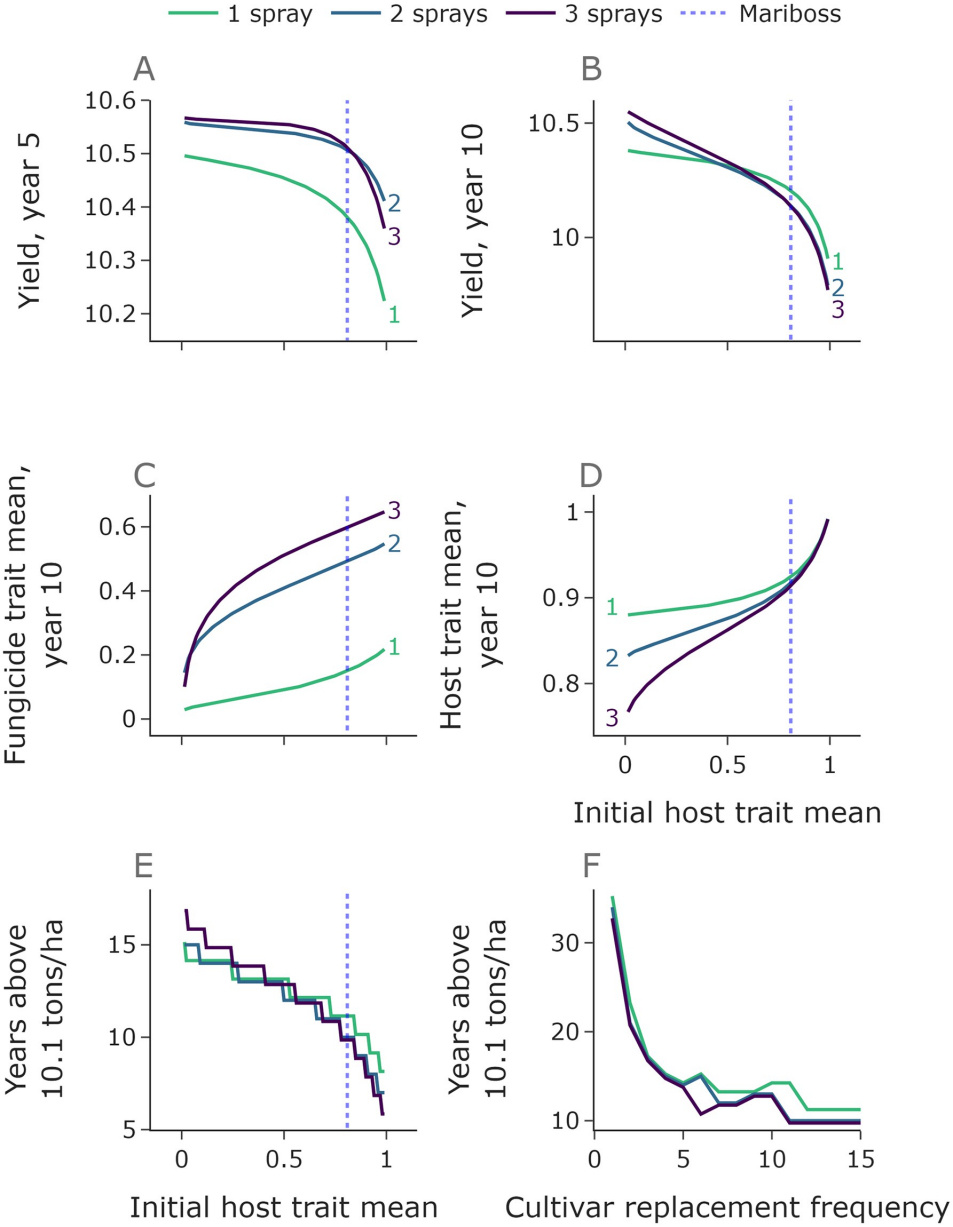
More effective cultivars protect fungicides more strongly and more frequent replacement leads to effective control for longer. In panels **A**-**E** we vary the initial host trait mean whilst keeping the shape parameter fixed. The vertical dotted lines show the fitted trait mean value for ‘Mariboss’. Lower host trait means than this value represent hypothetical cultivars which are even more disease-resistant. Over a short timescale (5 years) more applications is preferable than only one (**A**). However, for longer timescales and initial trait values close to that of ‘Mariboss’ (dotted line) one application outperforms two and three. For a given number of fungicide applications per year, the stronger the host protection (lower trait values), the slower the fungicide resistance develops (**C**). For a given initial host strength, higher numbers of fungicide applications protects the host more effectively (**D**). One application keeps the yield above a threshold of 10.1 tons/ha for the longest for all initial host trait values greater than 0.56 (**E**). Each year value is an integer but we offset each line vertically by a small amount to show the ordering. If cultivars are replaced regularly then the yield remains above the threshold for much longer (**F**). In this panel the cultivars are assumed to have identical performance to ‘Mariboss’. Here *n*_*l*_, *n*_*k*_ = 100.

For very short timescales (5 years), more fungicide applications gives improved yield (Fig 6A). However over longer timescales more applications leads to reduced yield for all but the strongest of our theoretical host cultivars (Fig 6B). This is because selection for fungicide resistance is stronger with more applications, although for particularly strong host protection higher numbers of fungicide applications are preferable due to increased mutual protection between host and fungicide. It should be noted that these particularly strong hosts have vastly stronger protection than the fitted cultivar ‘Mariboss’ (dotted vertical line).

Strategies with more fungicide applications lead to faster selection for resistant strains and correspondingly a higher fungicide trait mean in year 10 (Fig 6C). Stronger host protection (lower initial host trait mean) leads to lower values of the fungicide trait mean due to the protection offered by the cultivar to the fungicide via reducing disease pressure. Similarly, the host trait mean after 10 years of the integrated management strategy is reduced when more fungicide applications are used (Fig 6D), since more intensive fungicide application programmes offer stronger protection to the host. This difference is less exaggerated for cultivars with efficacy comparable to ‘Mariboss’ than for hypothetical ones with extremely low initial trait means.

As an overall summary of the durability of the combined programme, we compared the number of years for which yield remains above a threshold of 10.1 tons/ha. This threshold value was chosen as a value slightly higher than the yield achieved with a resistant cultivar but no fungicide input (9.98 tons/ha). One application outperforms two and three for all host trait values above 0.56 (Fig 6E), although note that this result depends on the choice of threshold (for very high yield thresholds one fungicide application is not sufficient to achieve a high enough yield even in year one).

We ran a second scan where we assume that the cultivar is replaced at regular intervals, from every year to every *N* years (Fig 6F). In practice this would correspond to wheat breeders releasing new varieties to market. Each time the current cultivar is replaced with another cultivar with efficacy matching that of ‘Mariboss’, but to which the pathogen population has not yet adapted to cultivar resistance. This prolongs the effectiveness of the host protection and increases the protective effect offered by the host to the fungicide. For very regular replacement (replacement frequency 1 or 2), the number of years above the threshold is much higher than without replacement. However, it should be noted that the cost of producing new disease-resistant cultivars can be very large (and take many years), so very regular replacement may be unrealistic in practice. The jagged nature of the lines is caused by the hard 10.1 threshold—for example, there is a sharp jump between 10 years and 11 years for 3 sprays, because without any replacement the strategy would fail in 10 years but if replacement happens in the final (10th) year then the sudden improvement in cultivar control leads to several more years of acceptable yields. With a replacement frequency of 5 years we get replacement in the 5th and 10th years before failure, but with a replacement frequency of 6 years the strategy fails in the 11th year just before the second replacement, so the outcome is significantly worse.

## Discussion

Integrated disease management strategies will be required for sustainable control of STB [53]. Many such strategies involve combining chemical control with resistant cultivars. Pathogen evolution poses a severe threat to the effectiveness of fungicide and cultivar control of crop diseases, meaning that understanding how to optimally combine these disease management strategies will be crucial to maintaining strong crop yields. Although many fungicides currently in use are challenged by quantitative pathogen resistance (for example the azoles), the model presented here is the first model of quantitative fungicide resistance to be fitted to field data (Fig 2). Further, the model incorporates both cultivar and fungicide control.

Our analysis shows that more fungicide applications leads to higher levels of resistance in the pathogen population (Fig 3). However, for any starting distribution of pathogen trait values, more fungicide applications give greater control in the first year. This trade-off affects which application strategy is optimal. Over short time frames, high numbers of fungicide applications lead to improved disease control. However, over longer timescales reduced numbers of applications are preferable since they reduce the selection for resistant strains and eventually this leads to higher yields. Note that development of new fungicide groups requires huge investment and takes a long time, and under the current European green-deal may not be feasible in Europe [54]. This means that the threat of resistance in the long term is highly significant and costly when compared to reduced yields in the short term. Another consideration is how risk-averse the grower is—in particularly high disease pressure years the improved control offered by higher numbers of application can be notable (Fig 4), but repeatedly using 3 sprays causes resistance to develop rapidly. One approach could be to use more applications in the highest disease pressure years but in general use the minimum number of applications possible to achieve a desired level of disease control, perhaps with the aid of a decision support system [54].

The model shows that disease control offered by deployment of disease-resistant cultivars is an effective way to reduce disease severity and increase yield (Fig 5). There is an added benefit in using resistant cultivars that it delays fungicide resistance development (Fig 5D). This is caused by the cultivar reducing the pathogen infection rate. By reducing the infection rates of all strains, the difference between per capita infection rates is reduced, thus reducing selection according to the so-called ‘governing principles’ [21]. Similarly, fungicide applications can delay loss of cultivar control, in agreement with [22]. The more effective the cultivar, the more exaggerated the mutual protection effect (Fig 6). Again, over a shorter timescale higher yields are obtained with more fungicide applications per year, but over a longer timescale the outcome improves with fewer fungicide applications per year (Fig 6C and 6D). Frequent replacement of disease-resistant cultivars could be an effective way to maintain disease control (Fig 6G). For simplicity, the effect of host plant resistance on each pathogen strain was considered to be constant throughout the season. Future work may address how ontogenic resistance [55] may influence disease management strategies, which have previously been explored in the case of powdery mildew on grapevine [28]. The results in this paper would suggest that applying fungicide doses at times during a growing season when host plant resistance is maximal would result in reduced selection for resistance.

Interestingly the results suggest that increased fungicide input is less destructive as a strategy in the case of quantitative resistance than previous work would suggest is the case for qualitative resistance [26, 28]. This is likely because some level of control can be obtained when quantitative resistance is present, whereas once qualitative resistance arises control is essentially lost. This is evidenced by the more gradual decline in control in Fig 2D than is typically observed in the qualitative resistance case [28]. However, it should be noted that we did not have data on the relative efficacies of 1 vs 2 vs 3 spray programmes. Data comparing the control attained for fungicide strategies implemented over many years using different numbers of sprays would be required to confirm the model results, which still suggest increased sprays is worse for resistance management and for yield in the long run. Note that the resistance risk varies depending on the mode of action as classified by the Fungicide Resistance Action Committee [56]. Our findings agree with their recommendation that reducing fungicide input results in reduced selection for resistance. Future work could also explore the effect of varying fungicide dose.

There are various modelling assumptions that were used for simplicity or due to a lack of available data. We neglect modelling fitness costs [57], since we did not have data to inform how different strains might carry fitness penalties. STB has a latent period within which infection cannot be transmitted [58], which for simplicity is not modelled here. For simplicity, we also do not consider removal of infected tissue. However, we show that this simplification has essentially no effect the model results (S3 Text). We also assume that the infection rate is fixed within each season. A more complex model could include temporal variation in infection rate within a single growing season, whereas we only change the infection rate value between different growing seasons. However, particularly when compared to the vast majority of fungicide resistance models [24, 26, 28, 36, 46] in which environmental stochasticity is not considered at all, we contend this is a sensible treatment.

In practice, environmental stochasticity affects the decisions made by growers; in high disease pressure years higher fungicide input is required than in lower disease pressure years to achieve adequate control. Although we neglect to model this change in grower behaviour, future work could address the impact of different disease management decisions based on perfect or imperfect knowledge of disease pressure in each year. In Fig 4 we can see that the greatest changes in yield in early years comes in the most extreme years, i.e. when the disease pressure is the highest. By targeting 2 or 3 spray programmes in these high pressure years only (i.e. reducing fungicide sprays in other years), growers could avoid the biggest losses in yield whilst reducing the resistance increase caused by consistently applying 3 sprays.

We follow many past modelling studies in using a fixed value for inoculum [24, 26, 28, 46] but in general inoculum does vary. Further the type of inoculum (ascospores vs pycnidiospores) can have complex effects on the latent period and the dynamics over multiple seasons [59]. However, as shown in Fig 3, as resistance increases disease severity increases. Increased severity would correspond to a larger pool of inoculum, further increasing the disease pressure. Therefore we might expect an effect where higher numbers of sprays initially gives an additional benefit of reducing the pool of inoculum. However, as resistance (and disease severity) increases there would be increased inoculum which would further increase disease severity. This effect could exaggerate the benefit of 3 sprays over short timescales and the disadvantage over moderate/longer timescales.

Further, we had no data to inform exactly how mutation should be parameterised in the model, for example whether the mutation scale should depend on time or on the trait value. The latter would mean that more resistant strains would be less/more likely to produce offspring with similar trait values. Further, target site changes contributing to azole resistance in *Z. tritici* have interacting effects [60], which we neglect to model. Modelling epistatic effects adds extra complexity, since mutations would have to alter the phenotype (i.e. fungicide trait value) depending on the genetic background in which the mutations occur [60]. Validating such a model would require extremely detailed genetic data describing the probability of each phenotype having offspring with each other phenotype. All of this data would need to be in a form that could be mathematically related to the fungicide trait value in the model. Instead, we used a Gaussian mutation kernel, following the default choice from the theoretical model of quantitative cultivar resistance [35] and from theoretical population genetics [61]. However, other mutation kernel choices could have been used, for example exponential-power kernels [35, 62].

Previous theoretical work has suggested that emergence times may have a non-monotone response to fungicide response due to fewer mutations occurring in smaller pathogen populations [39]. However, although this effect is explicitly captured by our model (Eqs 12 and 17), we find that increasing the number of fungicide sprays leads to faster development of resistance. This is because the strength of selection by each fungicide spray plays a much more significant role than the change in mutation rates. Further, the difference in population size between different spray numbers is not that exaggerated since there are times where the pathogen grows in the absence of fungicide in all spray programmes, and the fungicide is modelled to slow the increase of infection rather than actively reducing the amount of infection. Future work could test these assumptions and/or incorporate demographic stochasticity, so that highly resistant pathogen strains at very low densities might randomly die out rather than persist.

We also assume that fungicide resistance is characterised solely in terms of the fungicide dose response curvature parameter (*θ*). We follow [24, 28] in using a fixed asymptote parameter value *ω* = 1, so that the pathogen growth rate at a theoretical infinite dose is 0 (N.B. due to the modelled decay in fungicide concentration this would not correspond to 0 severity). Future work could consider exploring whether the results change if a different asymptote parameter is used, or if resistance is instead characterised in terms of the fungicide asymptote parameter (*ω*), or a combination of both curvature and asymptote parameters. For a theoretical model of partial qualitative fungicide resistance, the time for resistance to emerge always decreased for higher fungicide doses when partial resistance was characterised in terms of the asymptote parameter (*θ*) [27]. However, when resistance was characterised in terms of the curvature (*θ*), the response was non-monotonic with respect to dose for some parameterisations [27]. This was caused by convergence at high doses of dose-response curves for resistant and sensitive strains. Future work could explore the effect of dose on resistance development in the quantitative resistance setting, although fungicide decay in the model presented here may reduce the impact of dose-convergence.

Finally spatial effects are ignored, despite promising early studies showing the potential of fungicide application patterns based on spatial risk [63]. This theme of spatial deployment is particularly well established in the parallel host plant resistance literature [29, 30, 64]. For simplicity, most of the theoretical fungicide modelling literature neglects modelling space [24, 25, 27, 28, 36, 39]. We introduce significant complexity by trying to model quantitative resistance over a continuous spectrum of pathogen strains, rather than a small number (2 or 4 in most papers [24–28, 36, 39]). As such, we omitted spatial considerations from this work. However, there is certainly scope to extend this model in future to consider spatial effects, in order to address questions about spatial spread of resistant strains and/or spatially targetted disease management strategies. This would provide another means of delaying fungicide resistance, by reducing the number of fungicide applications in all but the highest disease pressure locations.

We have also implicitly assumed the pathogen strain composition in the inoculum is set by the grower’s own actions in previous seasons, which is equivalent to assuming a population of growers who all act identically and who all suffer the consequences of their actions. In practice, of course, there is more complexity and growers can be incentivised by economics to both prioritise or deprioritise resistance management [65, 66]. Although ignoring these effects is a very common simplification in modelling studies, we note game theory [67] is an attractive framework to go beyond this.

The model fitting process was somewhat limited by the nature of the data available. Ideally we would have data on both the response of isolates to fungicide and cultivar control as well as the frequencies of these isolates in the pathogen population over many years. Further, to incorporate fitness costs into the model, we would need data on the relative infectivity of these isolates in the absence of cultivar control. For azole resistance, this may be difficult to characterise due to the complexity of the CYP51 fitness landscape [68]. Future work could compare how the model results differ for various hypothetical fitness landscapes, for instance by letting the infection rate of the pathogen strains depend on *k* (e.g. testing the model results if the infection rate linearly decreases in *k*). Although EC50 values have been recorded for different pathogen isolates [69], we did not have sufficiently many years of these data available from the same experimental setup to support the model fitting process. Further, the mapping from experimentally measured EC50 values of pathogen strains to the effect of the fungicide on those strains’ infection rates is not yet characterised. Although we were able to fit the model based on the decline in disease control, we had to infer the initial pathogen distribution and use the final few years of data for model validation. Recent research shows that most genotypic diversity is found within individual fields [48]—so field-averaged control data is sub-optimal for our purposes. Ideally we would have data to describe the pathogen distribution from the first year the mode of action appeared on the market. We could then use this to show that the model predicts a decline in control comparable to that observed in the field. If we had access to data on the full distribution in successive years, the model fitting would become more powerful.

The model could be extended to address other disease management questions. Many of these future lines are obvious extensions now we have a fitted model of quantitative resistance in hand, given the long history and diverse applications of past models of qualitative resistance [24, 26, 28, 46]. We have considered full-dose applications of fungicide here; future work could include an analysis of dose choice and optimise for number of applications and dose in each application. A basic economic analysis could be included by accounting for the cost of each fungicide application [49, 70]. By extending the model to include two fungicides we could address questions on fungicide mixtures and whether mixtures outperform alternations in the case of quantitative resistance. We have considered strategies that are fixed in time, but it would be interesting to explore strategies that respond to the level of disease pressure and the level of resistance through time, e.g. something analogous to the so-called ‘Merry Dance’ [71]. There are many other crop diseases which are controlled by azole fungicides, for example powdery mildew, brown rust in wheat crops, citrus fruit mould and soybean rust [70, 72]. There are also pathogens (including *Z. tritici*) controlled by other fungicides to which resistance is quantitative, for example SDHI fungicides [17]. With appropriate data describing how yield of untreated plots compares to treated plots over many years, the model could be adapted to explore optimal management of these pathosystems, as well as accounting for combinations of chemical treatments. Many of these are questions that have been addressed, perhaps only partially, in the case of qualitative resistance [24, 26, 28, 36], but for which the modelling framework here offers the possibility of transferring to a more complex, but more realistic, setting.

## Supporting information

S1 Text Fitting initial inoculum. (PDF)

S2 Text Fitting mutation parameters. (PDF)

S3 Text Testing the mutation scale assumption and model structure. (PDF)

S4 Text Example distributions—Varying the mean but keeping the shape parameter fixed. (PDF)

S1 Data A CSV file relating disease severity values to yield values. (CSV)
