# Supplementary material for: Modelling quantitative fungicide resistance and breakdown of resistant cultivars: Designing integrated disease management strategies for Septoria of winter wheat
Source: PLoS Comput Biol. 2023 Mar 28;19(3):e1010969. doi: 10.1371/journal.pcbi.1010969 (PMC10081763; doi:10.1371/journal.pcbi.1010969)
Supplement: S1 Text — (PDF) [file pcbi.1010969.s001.pdf]

S1 Text

Fitting initial inoculum

We did not have data to describe the amount of inoculum (I_0) at the beginning of the modelled season ($t_{start} = T_1$), so we used disease severity data (Dataset A) for septoria severity from three time points (t_A, t_B and t_{end}, Table 2 – main text) within a single growing season to fit a value. We filtered the data so that we only considered plots untreated with fungicide, and trials for which the disease severity was greater than 0.

We used the freely-available Python package Optuna to sample potential values for the infection rate β_0 and initial inoculum I_0. For each pair of values, we found the squared distance between the logit disease severities from the model and from the data at each time point. The logit is defined as follows:

$$\text{logit}(p) = \log_{10}\left(\frac{p}{1-p}\right). \tag{1}$$

The logit scale was chosen since the disease progression is approximately linear on a logit scale and this choice means the severities from earlier in the year have a comparable impact on the model fit to those later in the year. The optimal parameters were the pair of values which minimised the sum of the squared distances (Text S1 Fig S1).

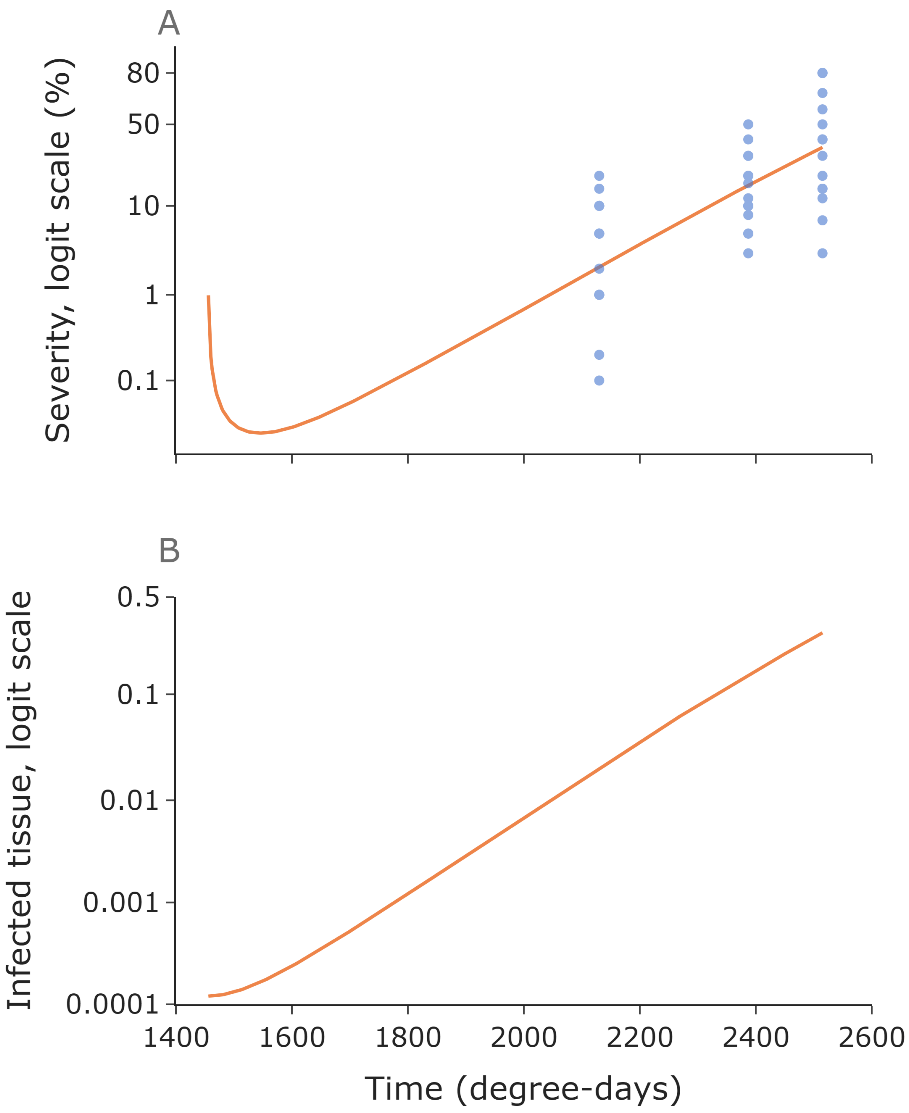

S1 Text Figure S1. Fitting I_0. We find the optimal values for initial inoculum I_0 and infection rate β_0 to fit the observed severity data. Initially the model severity decreases since the host grows faster than the infection [1, 2] (**A**). However, the amount of infectious tissue monotonically increases throughout the season (**B**). Note that infectious tissue is given as a percentage of the maximum amount of tissue after host growth is complete.

Instead of using the value of β_0 found by this process, we had a much larger dataset with severities at the end of the modelled season (growth stage 75) which allowed us to find a full distribution of infection rates to sample from which facilitated an exploration of the effects of environmental stochasticity, as described in the main text.

References

1. Ferrandino FJ. Effect of crop growth and canopy filtration on the dynamics of plant disease epidemics spread by aerially dispersed spores. Phytopathology. 2008;98:492–503. doi:10.1094/PHYTO-98-5-0492.

2. Bailey DJ, Paveley N, Spink J, Lucas P, Gilligan CA. Ecology and epidemiology epidemiological analysis of take-all decline in winter wheat. Phytopathology. 2009;99:861. doi:10.1094/PHYTO-99-7-0861.
