# Supplementary material for: Modelling quantitative fungicide resistance and breakdown of resistant cultivars: Designing integrated disease management strategies for Septoria of winter wheat
Source: PLoS Comput Biol. 2023 Mar 28;19(3):e1010969. doi: 10.1371/journal.pcbi.1010969 (PMC10081763; doi:10.1371/journal.pcbi.1010969)
Supplement: S2 Text — (PDF) [file pcbi.1010969.s002.pdf]

S2 Text

Fitting mutation parameters

[1] estimate that wheat fields typically produce 2.3 to 10.5 trillion pycnidiospores per hectare, of which between 28 and 130 million spores carry adaptive mutations to counteract fungicides and/or resistant cultivars. This allows us to produce an estimate for the proportion of offspring which carry a mutation by dividing the average number of spores carrying mutations by the average total number of spores:

$$p_M = \frac{0.5 \times (28 + 130) \times 10^6}{0.5 \times (2.3 + 10.5) \times 10^{12}} \approx 1.23 \times 10^{-5}. \quad (1)$$

Finding a value for the mutation scale parameter was more complicated. We chose to consider the scenario with no standing variation, meaning that the initial pathogen population consisted of a single pathogen strain and mutation was the sole driver of loss of control. This gives us an upper bound for the mutation scale. In general we expect control breakdown to be caused by a combination of selection for existing resistant strains and well as on strains that arise due to mutation.

We used the same dataset for fungicide control as used in the fungicide fitting process described in the materials and methods section (Dataset C). We only considered the first and last years since we were seeking an upper bound for the mutation scale, meaning that the overall loss of control over the full timescale was more important than a close match to the shape of the control breakdown curve. The shape of the breakdown curve depends on the initial trait distribution, but we were most interested in showing that the model would lead to a sufficiently rapid loss of control for the appropriate choice of mutation scale. We used the fitted value from the fungicide control data [2] directly rather than using the standard error values to generate distributions of values since we were only interested in the magnitude of the breakdown from the first to the final year.

We used the open-source Python package Optuna to find the optimal initial trait value and mutation scale (Text S2 Fig S1). The optimal parameter values were those that led to the minimal sum of squared residuals between the control values from the model and the data in 2001 and 2018 (first and last years).

For all other model runs we assumed that some of the breakdown of control was caused by initial standing variation in the pathogen population, and some was caused by mutation of the pathogen population. The mutation scale used in the model was 10% of the upper bound found in this section, arbitrarily chosen due to the lack of data to determine a value. The 10% assumption/choice is tested in Text S3. We also assumed that the host mutation scale is the same as the fungicide mutation scale. The mutation parameter values are shown in Text S2 Table S1.

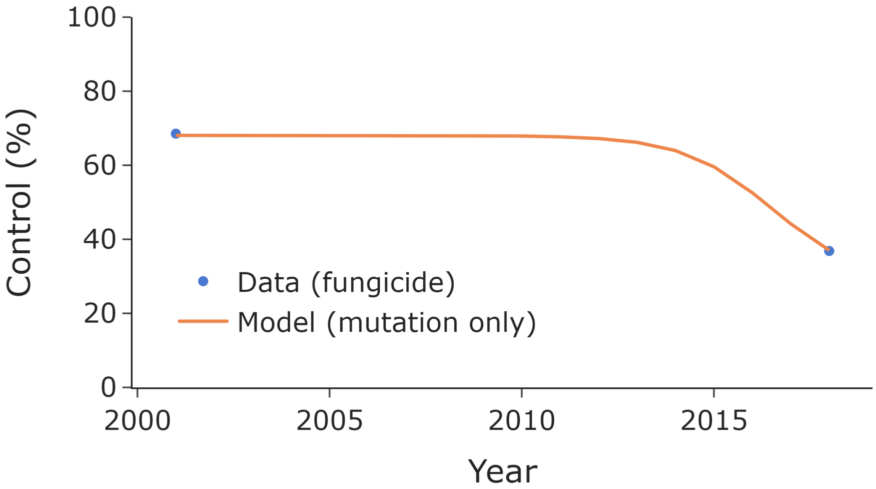

S2 Text Figure S1. Mutation scale fitting. We found the optimal initial trait value and mutation scale that led to the model matching the level of control achieved in the first and last year. The initial trait value determined the level of control initially, and the mutation scale affected the rate of loss of control. The resulting mutation scale is shown in Text S2 Table S1. The value found is an upper bound for the mutation scale, since this is the value in the scenario where there is no initial standing variation in the pathogen population. All other model runs involve initial standing variation and a lower value for the mutation scale (assumed to be 10% of the upper bound), as per the model fit in the main text (Fig 2). Here $n_k = 500$.

**Parameter**	**Value**
Mutation proportion	1.23×10^{-5}
Mutation scale upper bound	0.0198
Mutation scale used in the model	0.00198

S2 Text Table S1. Mutation parameters used in the model, reported to 3 significant figures.

References

1. McDonald BA, Suffert F, Bernasconi A, Mikaberidze A. How large and diverse are field populations of fungal plant pathogens? The case of *Zymoseptoria tritici*. Evolutionary Applications. 2022;15:1360–1373. doi:https://doi.org/10.1111/eva.13434.

2. van den Bosch F, Blake J, Gosling P, Helps JC, Paveley N. Identifying when it is financially beneficial to increase or decrease fungicide dose as resistance develops: An evaluation from long-term field experiments. Plant Pathology. 2020;69:631–641. doi:10.1111/PPA.13155.
