# Supplementary material for: Modelling quantitative fungicide resistance and breakdown of resistant cultivars: Designing integrated disease management strategies for Septoria of winter wheat
Source: PLoS Comput Biol. 2023 Mar 28;19(3):e1010969. doi: 10.1371/journal.pcbi.1010969 (PMC10081763; doi:10.1371/journal.pcbi.1010969)
Supplement: S3 Text — (PDF) [file pcbi.1010969.s003.pdf]

S3 Text

Testing the mutation scale assumption and model structure

Here we test the assumption that the mutation scale was 10% of the upper bound mutation scale value found in Text S2. We test 5 possible values: 0%, 1%, 10%, 50% and 90%. For each value, we fit a fungicide distribution, finding the optimal parameters using the Optuna package (as in the main text).

We also test the model when removal of infectious tissue is included. This changes the fungicide only model equations (Eqs 14, 15). The S equation is unchanged:

$$\frac{dS(t)}{dt} = g(t) - \Gamma(t)S(t) - S(t)\overline{\beta I}(t), \tag{1}$$

but the I equation gains an extra term $-\mu I(k,t)$:

$$\frac{dI(k,t)}{dt} = S(t)G(k,t) - \mu I(k,t) \qquad \text{for } k \in [0,1]. \tag{2}$$

Ignoring removal of infected tissue was a model simplification which avoids the need for the extra removal rate parameter μ. The within-season dataset (dataset A) was too small and too noisy to be able to simultaneously convincingly infer values for I_0 (initial inoculum), β (infection rate) and μ (removal rate). However, in a simpler model of monogenic resistance fitted to the same pathosystem the precise model structure was found to have very little effect on the strategy recommendation [1]. Here we adopt the removal rate value $mu = 1/456$ (degree-days^{-1}) used in [1]. Note that the [1] model was fitted to data from the UK rather than Denmark, and so a 'full' refit of the model would perhaps take a slightly different value for this parameter.

We find that the model results are virtually identical regardless of whether removal of infected tissue is included in the model structure or not.

Results

All values for the proportion of the maximum mutation scale achieve a comparable model fitting score on the training sets (Text S3 Fig S1), although the shape of the fits vary slightly in each case (Text S3 Fig S2). The model fitting scores on the test set vary slightly (Text S3 Fig S1, Text S3 Table S1). The best result comes from the 10% value (in the model without removal) used in the main text.

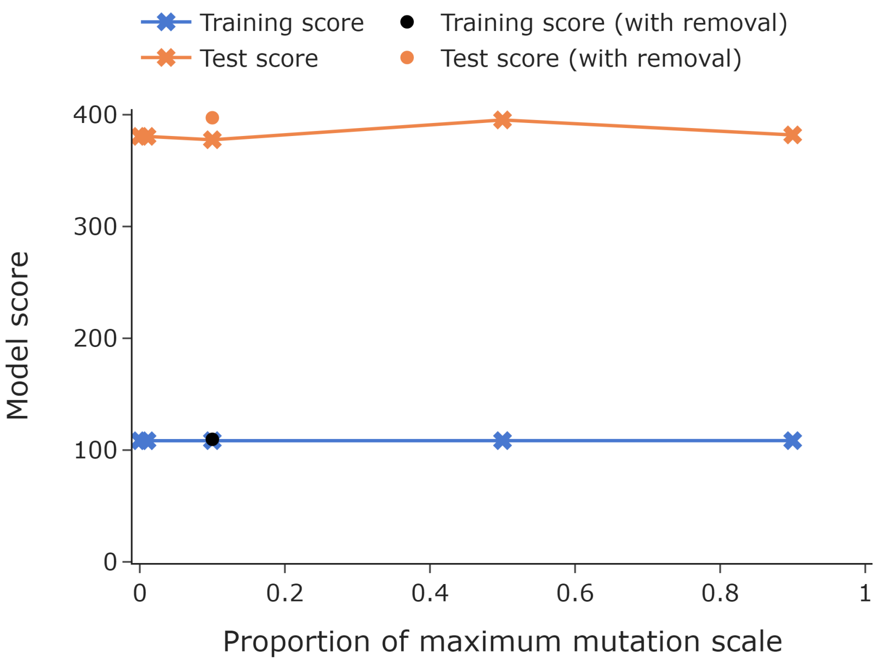

S3 Text Figure S1. Model fit scores. The model fit score here is the mean of the squared residuals between the model output and the fungicide control data for the optimal fungicide distribution parameters found using Optuna. We show the values on the training set (2001 to 2012) and on the test set (2013 to 2018). Lower values indicate a better fit. The performance on the training set is better than the test set, but all mutation scales lead to similar model performance. 'With removal' refers to the model fit with the modified model structure incorporating removal of infected host tissue (Text S3 Equation 2). The parameter values and model scores are shown in Text S3 Table S1.

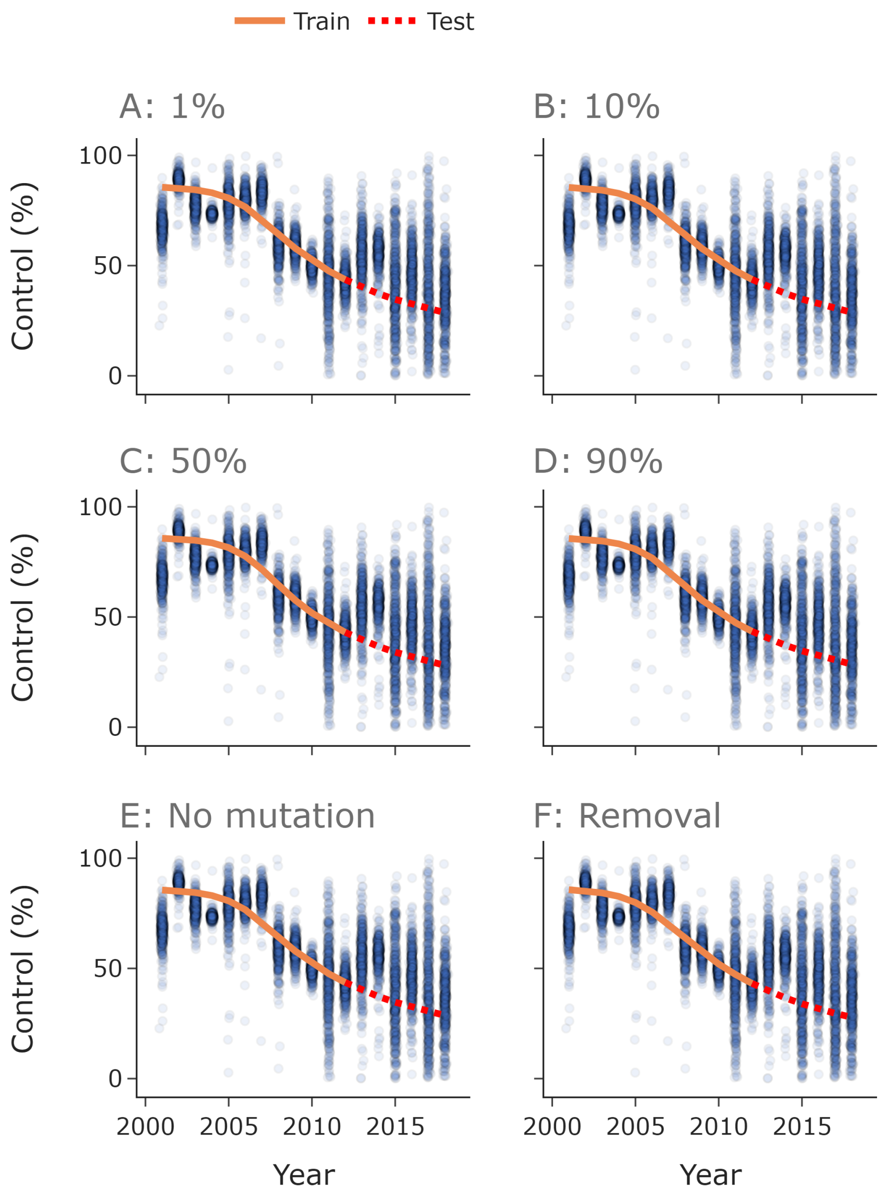

S3 Text Figure S2. Model fits. For each mutation scale (1%, 10%, 50% and 90% of the mutation upper bound from Text S2, **A-D**), we fit the model using Optuna. We also present the model fit without mutation (i.e. mutation scale is 0; **E**) and with removal of infectious tissue (**F**). Here $n_k = 500$.

The model outputs are qualitatively very similar (Text S3 Fig S3). This suggests that the value of the mutation scale used has very little impact on the model output or model results, assuming that the model is re-fitted to the data each time. Similarly including removal of infected tissue has very little impact on the model results. The optimal model parameters are quite similar across the different model fits (Text S3 Table S1).

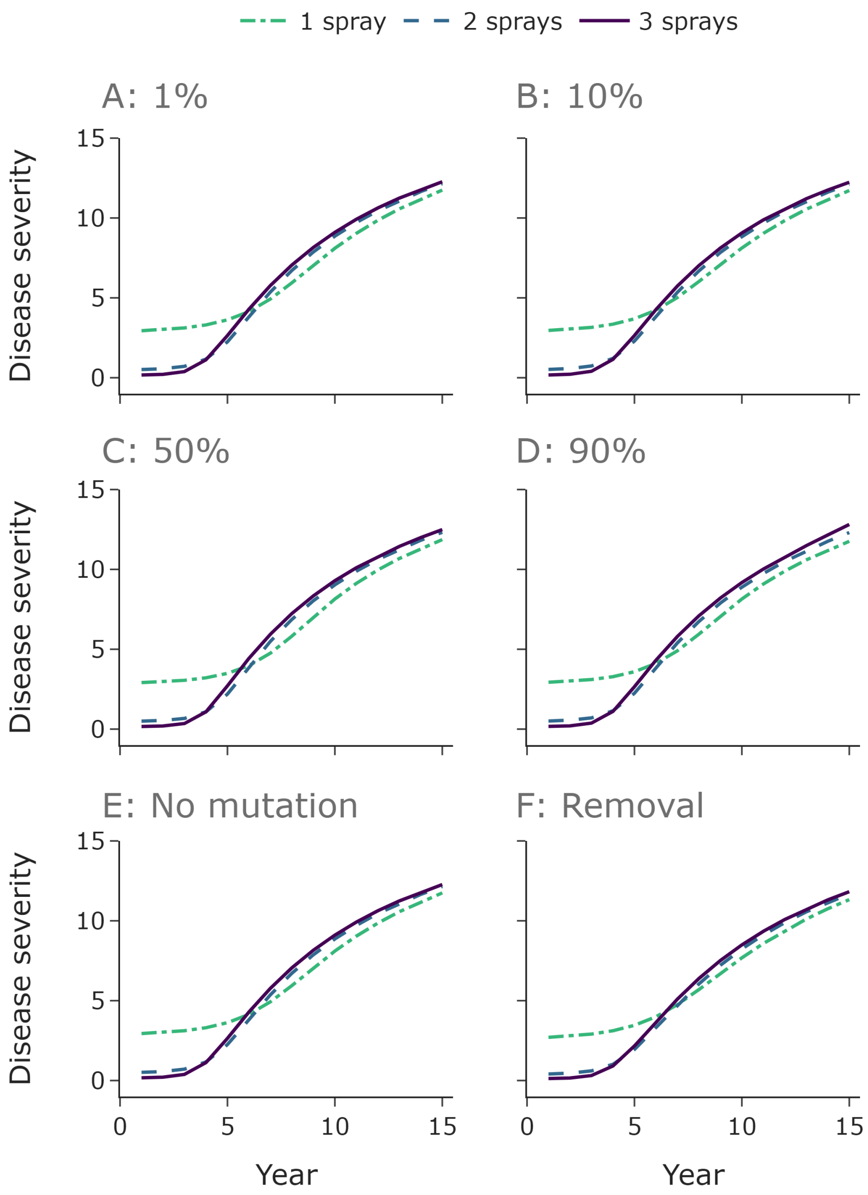

S3 Text Figure S3. Model outputs. The model outputs for all percentage values are qualitatively similar. Here we use the median value for the infection rate β_0 in every year. The parameter values are in Text S3 Table S1. Here $n_k = 500$.

% of mutation scale upper bound	Distribution mean	Distribution shape parameter	Training score	Test score
0%	9.69	0.816	108.38	380.58
1%	9.69	0.816	108.38	380.58
10%	9.44	0.843	108.44	377.61
50%	10.4	0.745	108.44	395.32
90%	9.84	0.801	108.45	381.91
10%, with removal	8.91	109.63	109.63	397.28

S3 Text Table S1. Optimal distribution parameters for each mutation scale, quoted to 3 significant figures except for training/testing scores which are quoted to 2 decimal places. The 10% value (without removal) we assumed in the main text gives the best performance on the test set.

References

1. Elderfield JAD, Lopez-Ruiz FJ, van den Bosch F, Cunniffe NJ. Using epidemiological principles to explain fungicide resistance management strategies: why do mixtures outperform alternations? Phytopathology. 2018;44:1–69. doi:10.1094/PHYTO-08-17-0277-R.
