# Supplementary material for: Modelling quantitative fungicide resistance and breakdown of resistant cultivars: Designing integrated disease management strategies for Septoria of winter wheat
Source: PLoS Comput Biol. 2023 Mar 28;19(3):e1010969. doi: 10.1371/journal.pcbi.1010969 (PMC10081763; doi:10.1371/journal.pcbi.1010969)
Supplement: S4 Text — (PDF) [file pcbi.1010969.s004.pdf]

S4 Text

Example distributions – varying the mean but keeping the shape parameter fixed

In Fig 6 (main text) we test how outcomes vary with different cultivar efficacies depending on the initial mean trait value. Text S4 Fig S1 shows some examples of the initial host distributions used, alongside the distribution corresponding default host trait mean used in the other model runs.

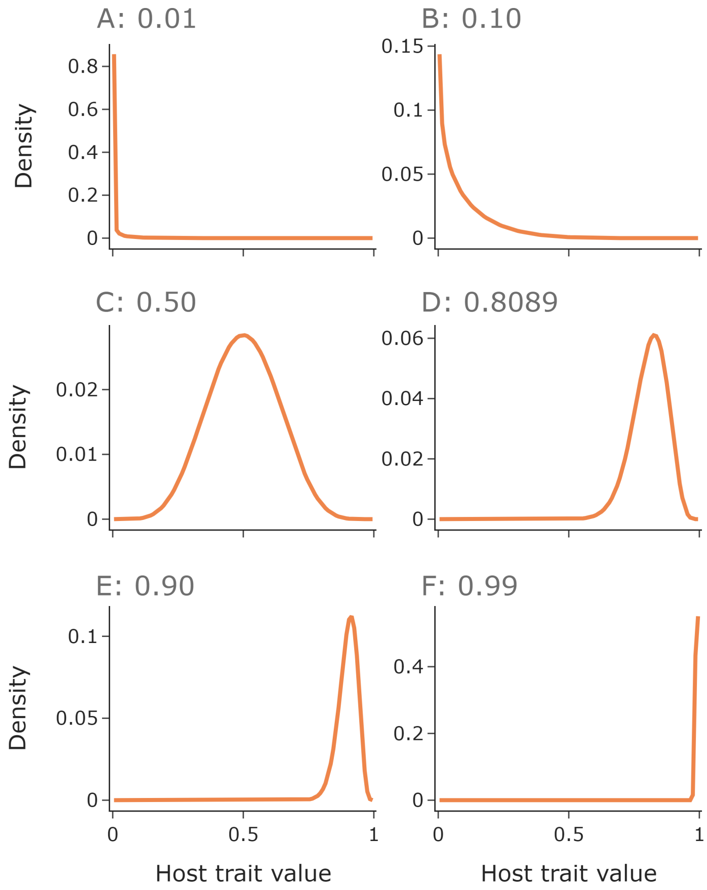

S4 Text Figure S1. Example initial host distributions used in Fig 6 (main text). We show 6 example host distributions. In Fig 6 the initial trait mean varies from 0.01 (shown in **A**) to 0.99 (shown in **A**). For comparison, we show the initial distribution for the default mean host trait value in panel **D**.
